# Understanding the genetics of neuropsychiatric disorders: the potential role of genomic regulatory blocks

**DOI:** 10.1038/s41380-019-0518-x

**Published:** 2019-10-15

**Authors:** Anja Barešić, Alexander Jolyon Nash, Tarik Dahoun, Oliver Howes, Boris Lenhard

**Affiliations:** 1 MRC London Institute of Medical Sciences, London, W12 0NN UK; 2 Institute of Clinical Sciences, Faculty of Medicine, Imperial College London, London, W12 0NN UK; 3 Department of Psychiatry, University of Oxford, Warneford Hospital, Oxford, OX37 JX UK; 4 Sars International Centre for Marine Molecular Biology, University of Bergen, N-5008 Bergen, Norway

**Keywords:** Neuroscience, Schizophrenia, Autism spectrum disorders, Bipolar disorder

## Abstract

Recent genome-wide association studies have identified numerous loci associated with neuropsychiatric disorders. The majority of these are in non-coding regions, and are commonly assigned to the nearest gene along the genome. However, this approach neglects the three-dimensional organisation of the genome, and the fact that the genome contains arrays of extremely conserved non-coding elements termed genomic regulatory blocks (GRBs), which can be utilized to detect genes under long-range developmental regulation. Here we review a GRB-based approach to assign loci in non-coding regions to potential target genes, and apply it to reanalyse the results of one of the largest schizophrenia GWAS (SWG PGC, 2014). We further apply this approach to GWAS data from two related neuropsychiatric disorders—autism spectrum disorder and bipolar disorder—to show that it is applicable to developmental disorders in general. We find that disease-associated SNPs are overrepresented in GRBs and that the GRB model is a powerful tool for linking these SNPs to their correct target genes under long-range regulation. Our analysis identifies novel genes not previously implicated in schizophrenia and corroborates a number of predicted targets from the original study. The results are available as an online resource in which the genomic context and the strength of enhancer–promoter associations can be browsed for each schizophrenia-associated SNP.

## Introduction

The primary aim of genome-wide association studies (GWAS) and other genetic association studies is arguably to serve as the first step in elucidation of the biological mechanisms responsible for the onset of disease, which will eventually lead to their translation into clinical practice. While GWAS and other genetic association studies have made great steps towards understanding many diseases, progress has so far fallen short of initial expectations for most neuropsychiatric disorders. This is primarily due to the difficulty of identifying the biological effect of variants identified as significantly associated with the disorder. More philosophically, it is becoming increasingly recognised that considering interactions across the whole system is required to understand mechanisms in biology [1], and this may be particularly the case for brain disorders [2]. In the rest of the article we will refer to GWAS because they are the focus of much genetic research in neuropsychiatric disorders at the moment, but the same issues apply to other genetic association studies.

The GWAS approach attempts to identify the statistically significant overrepresentation of specific single-nucleotide polymorphism (SNP) alleles in a group of affected individuals vs. a healthy control group. This is performed for a panel of hundreds of thousands of SNPs, selected such that they “tag” each linkage disequilibrium (LD) block across the genome at least once. Due to the LD structure of the genome, each identified variant will have hundreds to thousands of other variants in its proximity that are also significantly associated with the trait. The first stumbling block when interpreting GWAS results is thus the identification of the causal variant responsible for the statistical association of the tagged SNP and the trait being studied. The identification of the variant that underlies the biological effect responsible for the statistical association with the disease is known as fine mapping (reviewed in [3]). This procedure is highly dependent on the quality of genotyped data used for the reference (relatively large LD blocks present in the European population were historically especially problematic [4]), the size of the GWAS sample and the concordance of variant quality control across studies used [5], with notable recent success by Mahajan et al. [6] and others [7, 8].

**Fig. 1:**
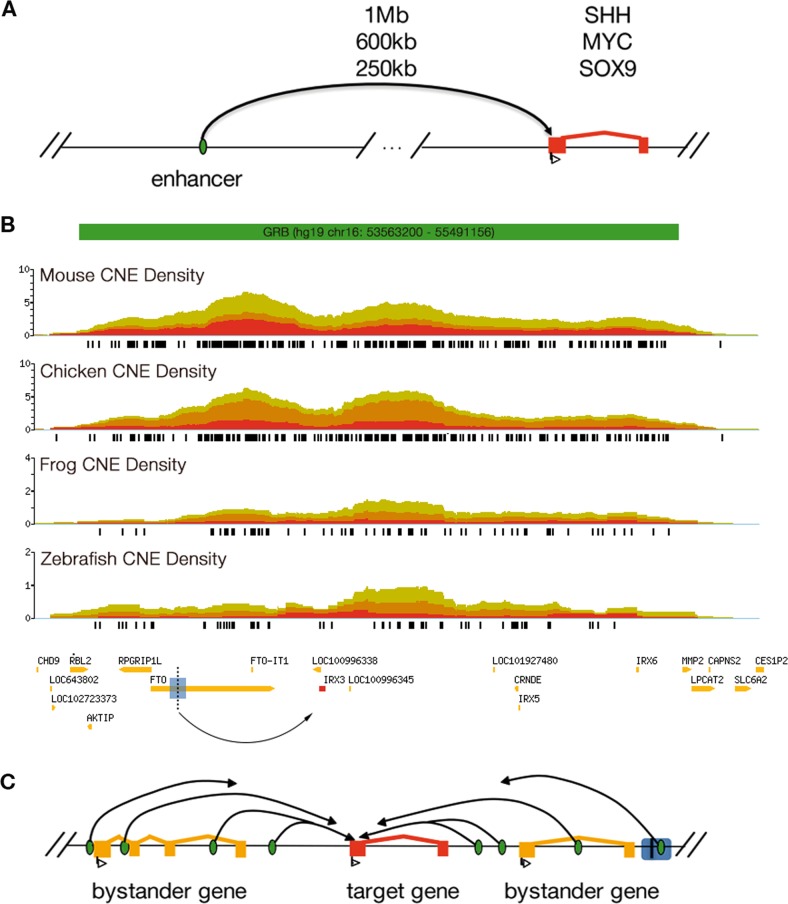
Long-range gene regulation. **a** An enhancer can target a gene over large genomic distances. **b** A genomic regulatory block (GRB) spanning a 1.9 Mb region of the human genome. This region displays high levels of non-coding conservation between the human and four vertebrate genomes, reflecting evolution over ~435 million years. The non-coding conservation peaks around *IRX3*, the predicted target gene in this GRB. The black vertical line and blue rectangle mark the genomic position of the obesity-associated SNPs (rs1421085 and rs9939609) from the Ragvin et al. study, and the linkage disequilibrium block around it, respectively. Each SNP falls within an enhancer that is capable of activating the expression of the *IRX3* gene, shown in red. **c** The GRB model: conserved non-coding elements (shown in green) within a GRB contact the target gene’s promoter (shown in red) through looping and regulate its transcriptional activity in multiple contexts. SNPs in the linkage disequilibrium with regulatory elements involved in long-range gene regulation are often erroneously assigned to the nearest bystander gene, associating the wrong gene to the disease phenotype

Even with knowledge of the exact causal variant responsible for a GWAS hit, there is often significant difficulty in identifying its biological effect, particularly in the case of non-coding SNPs. In the catalogue of published GWAS, ~95% of the ~47,000 identified SNPs (as of April 2018) fall in non-coding regions of the genome, and are enriched in regulatory elements [9, 10]. In many cases the identified variants fall within gene deserts, potentially millions of base pairs away from the closest gene. This makes interpreting their biological effect very difficult. Historically, the most prevalent practice in the literature is to assign a non-coding variant’s effect to the closest gene in terms of genomic distance. However, this approach neglects the three-dimensional structure of the genome, and fails to consider that many genes are subject to long-range regulation by enhancers up to a megabase away from their transcription start site, discussed in more detail in Box 1. This is often the case for developmental genes such as *SHH* [11], *MYC* [12] and *SOX9* [13], many of which are relevant to neuropsychiatric disorders. In each of these cases enhancers loop over large genomic distances to contact the promoter of their target gene, even in the presence of intervening genes (Fig. 1a). Thus, assigning SNPs that are part of regulatory elements to the genes nearest to their loci means their more distant targets may be missed and the wrong genes can end up being investigated for disease mechanism. These long-range regulatory elements are often grouped together on chromosomes in arrays known as genomic regulatory blocks (GRBs) [14–16]. GRBs have particular characteristics that can be used to identify them and their targets (see Box 1 for more details).

Ragvin et al. have applied the GRB model to successfully determine the regulatory targets of non-coding SNPs associated with type 2 diabetes and obesity [17]. For example, they re-examined an SNP initially linked to the *FTO* gene. Using the extent of non-coding conservation and the GRB boundaries between humans and zebrafish, the authors were able to predict the correct regulatory targets of conserved non-coding elements (CNEs) spanning the GWAS implicated LD blocks for three loci, leading to predictions that the SNP in the *FTO* region disrupted a conserved motif regulating *IRX3* (Fig. 1). These predictions were validated by transgenic reporter assays and implicated IRX3 in the development of type 2 diabetes and obesity for the first time. The mechanism underlying the link between *IRX3* and these conditions was since established using both 4-C and CRISPR-Cas9 based methods, which showed that the SNP in the *FTO* region de-repressed *IRX3* expression, leading to altered energy metabolism and increased lipid storage [18, 19]. This illustrates the potential value of the GRB model in identifying the targets of non-coding SNPs found to be associated with a disorder in a GWAS.

Recently, the complexity of the gene regulation has become more broadly appreciated [20, 21], and there have been efforts to look beyond the closest gene and at the broader genomic context of the locus to identify the target gene affected by the variant [8, 22–24]. They potentially affected genomic region around the SNP encompassing all genes to be tested for the effect is generally defined in two main ways: (i) by setting a fixed distance around every SNP [24–26], typically 0.5–1 Mb (in extreme case 2 Mb) region upstream and downstream of the disease-associated variant, or (ii) using topologically associated domains (TADs), which are based on prior chromatin interaction experiments [27]. The former approach uses no additional information, therefore making no assumption on which locus is likely to be involved in long-range genomic interactions and thus considers all genes within a set distance around every locus. The search space for the target gene(s) is consequently large, which increases the information load and the potential for false positives or negatives. In addition, the set distance is arbitrarily defined. In contrast, the second approach uses experimentally determined topologically associated domains to define the search space, based on the functional assumption that interactions between an enhancer and a promoter do not cross TAD boundaries. However, this approach will still evaluate every enhancer–promoter pair as equally plausible, while the GRB model approach identifies the most likely target gene of the long-range regulation in every block.

TADs are units of 3D genome structure that are megabase-sized in mammalian genomes, and mostly invariant across cell types and to a large extent across species [28]. TADs delineate regions that preferentially interact with themselves over other regions of the genome; in other words, the vast majority of genomic interactions start and end within the same TAD. Recently, several cases where the disruption of TADs results in the emergence of disease phenotypes have been identified. For example, disruption of TADs has been shown to be responsible for introducing de novo enhancer–promoter interactions, resulting in the mis-regulation of gene expression during limb development [29], for erroneous activation of proto-oncogenes causing acute lymphoblastic leukaemia [30] and finally TAD data in the developing brain shed new light on the neurodevelopmental disorders [27].

In addition, a large effort has been made to map the interactions between active promoters and the rest of the genome in many tissues and cell types, for example revealing tissue-specific aspects of genome architecture in hematopoiesis [31]. However, TAD identification from Hi-C data both necessitates a significant amount of starting material, which is often not available for neuropsychiatric disorders due to limited availability of biological material from human brains, and incurs large sequencing costs due to the depth required to sufficiently cover the spatiotemporal complexity of gene regulation, especially in human genome.

It has been recently shown that most TADs correlate well with the span of GRBs: the boundaries of TADs are well predicted by the boundaries of high density of conserved non-coding elements in GRBs, arguing that these are two manifestations of the same underlying regulatory phenomenon [32]. Thus, the GRB approach discussed above could also help identify gene targets linked to the structural organisation of the genome without the material requirements or costs of Hi-C. An additional advantage of the GRB model is the ability to provide a tissue-invariant classification value of how likely the gene is to be subjected to long-range regulation for all the genes found within the GRB, thus significantly narrowing the search space for targets of SNPs involved in long-range regulation.

In the next section, we illustrate the potential use of the GRB approach combined with functional enhancer activity and gene expression data to inform the interpretation of genetic association studies using the example of schizophrenia.

Box 1 Some of the best known examples of regulatory elements acting on genes across extreme distances include key developmental genes such as *SHH* [11], *MYC* [12] and *SOX9* [13] (Fig. 1a). Each of these cases serves to illustrate that enhancers can loop over large genomic distances to contact the promoter of their target gene, even in the presence of intervening genes.

In many cases the regulatory regions of genes under the control of long-range regulation coincide with clusters of extreme non-coding conservation [17]. These conserved non-coding elements (CNEs) are long stretches of non-coding DNA, which are deeply conserved in vertebrates [68–70] and function as enhancer elements, driving complex spatiotemporal gene expression patterns [70–72]. The requirement for regulatory elements to remain in cis with the gene that they regulate [73] has constrained the organization of the genome, resulting in long syntenic arrays of CNEs clustered around genes involved in the regulation of development [14, 15, 69]. These syntenic arrays, known as genomic regulatory blocks (GRBs), can be used to delimit the boundaries beyond which it is unlikely a non-coding SNP may have an effect on gene expression (Fig. 1b). Furthermore, each GRB tends to target a single gene or a few related genes, distinguishable from bystander genes in promoter structure, specific patterns of epigenetic modifications, tissue specificity and range of biological functions [16] (Fig. 1c).

Since the original detection of GRBs and the specific features of their target genes [16], a more robust, fully automated method for GRB and target gene prediction has been developed (Tan and Lenhard, in preparation, and Supplementary Methods). Briefly, GRBs are detected as clusters of conserved synteny occurring at a higher rate than expected for the two genomes compared (here: human vs. mouse), excluding those GRBs that do not overlap a protein-coding gene. A total of 1072 genomic regulatory blocks have been detected in this manner, spanning between 28 kb and 5.35 Mb in length (mean 882 kb). Next, all genes in GRBs are given a target prediction value, based on a random forest model trained on a set of target genes from Akalin et al., using a range of features that are the strongest indicators of genes amenable to long-range regulation, i.e. length of CpG islands, gene entropy and CNE density (for more details, check Supplementary Methods). Using a simple cut-off on the score that has been normalised within each GRB, all genes spanning a GRB are then classified either as bystanders or targets.

Several GWAS detected disease-associated variants within introns of bystanders in GRBs, where the disease phenotype fitted well with loss of function of the target gene, were reviewed by Becker and Rinkwitz [74], and references therein), arguing for these non-coding variants being involved in regulation of the GRB target genes. In particular, they provide the GRB context for the intronic SNPs in ELP4 (a bystander gene, a target is *PAX6*) linked to aniridia; SNPs in the bystander LMBR (with target *SHH* a megabase away) causing preaxial polydactyly; again *SOX4* being targeted by intronic SNPs in *CDKAL1* and *IRX3* being targeted by SNPs in introns of the FTO, both associated with type 2 diabetes; and implicate intergenic SNPs between two bystanders to be misregulating MEIS2, ultimately leading to myopia.

## Application of the GRB model sheds new light on schizophrenia-associated loci

Schizophrenia is a severe mental disorder that is among the leading causes of global disease burden, with a lifetime prevalence of 0.7% [33–35]. It is a highly heritable neurodevelopmental disorder, but its genetic basis remains elusive [36–39]. It is thought to be caused by the complex interaction between inherited genetic predisposition and environmental risk factors [39–41]; see Box 2 for a discussion on heritability estimates and detectable proportion. In 2014, Schizophrenia Working Group of the Psychiatric Genomics Consortium performed a large multi-stage schizophrenia GWAS of 36,989 cases and 113,075 controls [42], further referred to as the PGC GWAS dataset. One hundred twenty-eight SNPs statistically associated with schizophrenia were identified. These 128 SNPs were then merged based on LD resulting in 108 loci, 83 of which had never been implicated in schizophrenia before. Possible targets for the non-coding SNPs were then assigned to the closest gene, or all genes that fell within a locus. This set of loci provides the ideal dataset to illustrate the potential of the GRB model to provide further insights into variants in non-coding regions.

We identified GRBs based on conserved synteny between human and mouse and overlapped these GRBs with the 108 schizophrenia-associated loci identified by the PGC. In total 52 of the 108 loci overlapped a GRB, with four GRBs each overlapping two schizophrenia-associated loci. For each locus, we compared the target genes proposed by the original GWAS with the target gene for that GRB. The aim was not to invalidate the gene list proposed in the PGC study, but rather to identify additional, potentially more plausible target genes for loci that are not trivial to interpret. All 108 schizophrenia loci are summarised with regard to GRB overlap and GWAS-associated and GRB-associated target genes in Supplementary Table S1. In short, we found that our GRB-based approach pointed to at least one target gene originally mentioned in the PGC dataset for 25 schizophrenia-associated loci, while for the remaining 27 loci that overlapped GRBs an altogether different target was predicted (Table 1). In total, our method predicts 120 genes to be under long-range regulation in GRBs overlapping schizophrenia-associated loci.

**Table 1:** A table of loci from the PGC study [42] overlapping a genomic regulatory block, with target genes assigned to each locus by the GWAS study and by the GRB method

Locus coordinates	GWAS SNPs	GWAS genes	GRB target genes	GRB by stander genes
chr5:60499143–60843543	rs4391122	ZSWIM6	SMIM15	NDUFAF2,ZSWIM6,RPL3P6,C5orf64,RNU6-913P,RN7SKP157
chr2:200715237–200848037	chr2_200825237_I	AC073043.2 C2orf47 C2orf69 TYW5	SATB2	PLCL1,RNU7-147P,SATB2-AS1,SEPHS1P6,FTCDNL1
chr7:110843815–111205915	rs13240464	IMMP2L	LRRN3	IMMP2L,DOCK4,DOCK4-AS1
chr11:130714610–130749330	rs10791097	SNX19	ADAMTS15,NTM,OPCML,SPATA19,IGSF9B	ADAMTS8,BAK1P2,C11orf44,PPP1R10P1,SNX19,RN7SL167P,RNU6ATAC12P,NTM-IT,RNU6-1182P,OPCML-IT2,OPCML-IT1,MIR4697,JAM3
chrX:21193266–21570266	rs1378559	CNKSR2	KLHL34	CNKSR2,SMPX
chr18:52747686–53200117	chr18_52749216_D, rs78322266, rs9636107	TCF4	CCDC68	RAB27B,MAP1LC3P,RNA5SP459,TCF4,MIR4529,RPL21P126
chr3:180588843–181205585	chr3_180594593_I, rs9841616	CCDC39 DNAJC19 FXR1	SOX2	FXR1,DNAJC19,SOX2-OT,RNU6-4P,FAUP2,RPL7AP25,RN7SL703P,RNA5SP150,RN7SKP265,RPL7L1P8
chr2:57943593–58502192	rs11682175, rs75575209	FANCL VRK2	BCL11A	VRK2,FANCL,EIF3FP3,LINC01122,RNU6-508P,RNA5SP94,RNU1-32P,MIR4432,RN7SL361P,RNU6-612P,ATP1B3P1,PAPOLG
chr18:53453389–53804154	rs72934570, rs715170	TCF4*	CCDC68	RAB27B,MAP1LC3P,RNA5SP459,TCF4,MIR4529,RPL21P126
chr3:2532786–2561686	rs17194490	CNTN4	CNTN6	RN7SL120P,RPL23AP39,RPL21P17,RN7SKP144,CNTN4,CNTN4-AS2,DNAJC19P4,CNTN4-AS1,IL5RA
chr3:52541105–52903405	rs2535627	GLT8D1 GNL3 ITIH1 ITIH3	SEMA3G,TNNC1,NT5DC2	PHF7,NISCH,STAB1,SMIM4,PBRM1,RNU6-856P,RNU6ATAC16P
chrX:68377126–68379036	rs5937157	PJA1	EFNB1,FAM155B	STARD8,ACTR3P2,SERBP1P1,PJA1,HMGN1P35,LINC00269,CYCSP43,EDA
chr17:2095899–2220799	rs4523957	SGSM2 SMG6 SRR TSR1	SCARF1,RILP,TLCD2,SERPINF2,RTN4RL1,HIC1,MNT	SLC43A2,RN7SL105P,PRPF8,MIR22HG,WDR81,SERPINF1,SMYD4,RPA1,DPH1,OVCA2,MIR132,MIR212,SMG6,RN7SL624P,SRR,HNRNPA1P16,TSR1,SNORD91B,SNORD91A,SGSM2,METTL16
chr15:61831663–61909663	rs12903146	VPS14C*	RORA	NARG2,CYCSP38,RNA5SP397
chr22:42315744–42689414	rs1023500, rs6002655	CENPM CYP2D6 FAM109B NAGA NDUFA6 SEPT3 SHISA8 SMDT1 SREBF2 TCF20 TNFRSF13C WBP2NL	NFAM1	TCF20
chr2:146416922–146441832	chr2_146436222_I		ARHGAP15,ZEB2	KYNU,MTND6P11,MTND5P24,MTND4P22,MTND3P9,GTDC1,ZEB2-AS1,TEX41,RPL6P5,RNU7-2P,RPL17P12,PABPC1P2
chr1:243503719–244002945	rs10803138, rs77149735, rs14403, chr1_243881945_I	AKT3 SDCCAG8	ZBTB18	SDCCAG8,MIR4677,AKT3,FABP7P1,AKT3-IT1,RN7SL148P
chr3:17221366–17888266	rs4330281	TBC1D5	SATB1	TBC1D5,PDCL3P3,RAD23BP1,RNU6-138P
chr8:60475469–60954469	rs6984242	CA8*	TOX	RNU4-50P,RNA5SP267,SLC2A13P1,CA8
chr14:30189985–30190316	rs2068012	PRKD1	FOXG1	C14orf23,RNU6-864P,RNU11-5P,PRKD1,RNU6-1234P
chr4:23366403–23443403	rs215411	MIR548AJ2*	PPARGC1A,DHX15,SOD3	GBA3,CDC42P6,RFPL4AP3,MIR573,RN7SL16P,ATP5LP3,HNRNPA1P65,CCDC149,LGI2
chr7:110034393–110106693	rs211829	IMMP2L*	LRRN3	IMMP2L,DOCK4,DOCK4-AS1
chr7:131539263–131567263	rs7801375	PODXL*	PLXNA4,LRGUK	CHCHD3,EXOC4,COX5BP3,SLC35B4
chr1:177247821–177300821	rs6670165	FAM5B	ASTN1,BRINP2,SEC16B	PAPPA2,PTP4A1P7,MIR488,RASAL2-AS1,RASAL2
chr1:207912183–208024083	rs7523273	C1orf132 CD46 CR1L	PLXNA2	C1orf132,CD34,RPS26P13,ATP5G2P1,MIR205HG
chr12:92243186–92258286	rs4240748	C12orf79*	BTG1	C12orf79,RPL21P106
chr12:103559855–103616655	rs10860964	C12orf42	PAH,ASCL1	IGF1,LINC00485,RNU7-184P,C12orf42

Only a subset with completely novel target genes by the GRB method proposed is presented here; for the full set of loci from the PGC dataset, see Supplementary Table S1

*Denotes nearest non-overlapping gene to the locus, as defined in the Supplementary Table S3 in [42]

**Fig. 2:**
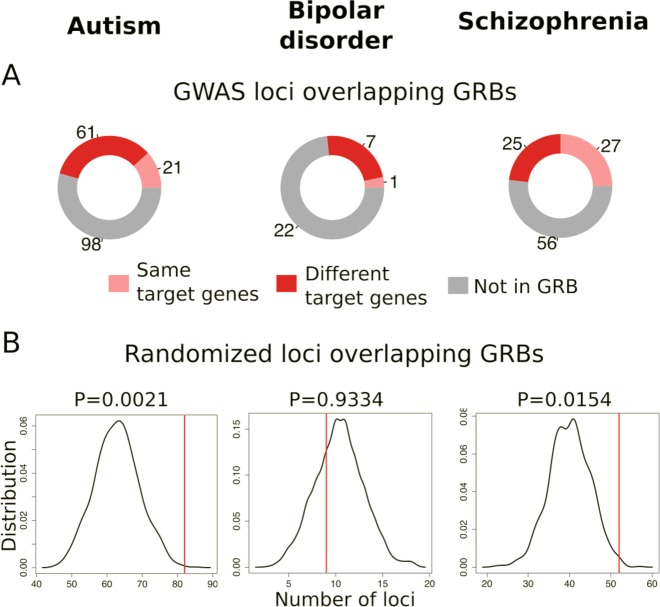
Long-range regulation in neurodevelopmental GWAS loci. **a** LD blocks that do not overlap GRBs are shown in grey. Loci in which the predicted GRB target gene was identified as schizophrenia associated in the original GWAS are shown in light red, and the loci in which the GRB model provides novel target gene predictions are shown in dark red. **b** The distribution of overlaps with the genomic regulatory blocks of the same number of random regions. *P*-value calculated based on *N* = 10,000 randomisations

We applied the same approach to the recent GWAS for autism and bipolar disorder, two other neuropsychiatric disorders with high heritability (Box 2). This analysis shows significant enrichment for the GWAS loci to overlap with GRBs in autism (*p* < 0.05) but not bipolar disorder (*p* > 0.9; Fig. 2b). In autism 82 of the 180 loci overlap a GRB, and the GRB-based analysis indicates different gene targets than those originally assigned for 61 of these loci (Fig. 2a). Thus, the GRB method could help identify novel gene targets from the GWAS studies of a number of neuropsychiatric disorders, although potentially not for bipolar disorder.

While both the vicinity of the non-coding SNP to a gene, and the GRB target gene prediction approach provide one or more putative genes through which a non-coding SNP may contribute to disease emergence, neither should be used as a definitive argument for the gene to be considered the SNP target. We appreciate that it would be informative to consider the effect of SNPs on all the genes within a GRB/TAD, but since the experimental validation of each enhancer–promoter pair is prohibitively expensive in terms of experimental time and resources, it is useful to focus on the most likely targets first. Thus, we include an additional metric of how responsive each of the candidate genes (GWAS-proposed genes and GRB target genes) is to the regulatory element(s) within disease-associated locus. The FANTOM5 consortium produced cap analysis of gene expression data (CAGE) for over a thousand human tissues and cell lines, assaying the exact position and quantity of transcription across the genome. The first advantage of this dataset that make it particularly amenable to investigating developmental aspects of schizophrenia is that unlike some of the recent schizophrenia-associated RNA-seq datasets (used in [23, 24]) and the GTEx project [43], FANTOM5 has transcriptional profiles of tissue, including neural tissue, from 75 foetal and newborn subjects. Secondly, it was found that enhancers undergo non-productive transcription initiation in contexts in which they are active [44], making it possible to capture the expression of a gene with the activity of all of the enhancers in its vicinity using a single CAGE experiment. For each GRB, we identify those genes whose activity, across a wide panel of tissues and cell lines, correlates best with the activity of its surrounding enhancers. These are presumably the genes that are most responsive to the activity of the enhancers and thus represent likely target genes of GRB regulation. For putative enhancers that are in high LD with schizophrenia-associated variants, this provides us with the most likely affected gene.

We have applied these methods to all loci across the genome. Here, we present four examples based on the analysis of the schizophrenia loci, which serve to illustrate specific situations in which considering variants in the GRB context is particularly effective in either providing novel hypotheses or refining existing ones. For the full analysis of the genomic context and enhancer–promoter pair expression correlations (for loci overlapping GRBs), and basic information on the other loci detected in the schizophrenia GWAS [42], we provide a Shiny [45] web app at http://scz.genereg.net/.

Box 2 Neurodevelopmental disorders like schizophrenia, autism and bipolar disorder display high heritability estimates, while at the same time only a small fraction of this heritability can be recapitulated through genome-wide association studies [75]. More specifically, heritability of schizophrenia has been estimated to be between 60% and 80% by large population-based studies and meta-analysis of twin studies [76, 77]; between 64% and 91% for autism by the most recent meta-analysis on twin studies [78] and between 60% and 80% for bipolar disorders [79]. On the other hand, the SNP-based heritability estimated on liability scale has been estimated between 23% and 32% for schizophrenia [52, 80, 81], 17% for autism and 25% for bipolar disorder [81].

This missing heritability may come from variants with smaller effect sizes thus far missed due to the sheer population size needed to achieve statistical significance when testing for small effects [36], or other types of genetic variation, such as copy number variants [38, 82, 83]. The GRB model is disease-agnostic and broadens the search space for target genes based on conserved synteny. This enables the detection of previously unidentified target genes, thereby implicating novel gene families or mechanistic pathways. By searching the regions surrounding other members of these enriched gene families or pathways for variants that appear suggestively associated but do not pass the genome-wide significance threshold, it may be possible to resolve a portion of the missing heritability, while also identifying potential new therapeutic targets.

While the rest of our analysis focuses exclusively on schizophrenia-associated variants, two additional recent datasets of SNPs associated with other neurodevelopmental phenotypes, i.e. autism spectrum disorder (ASD) [84] and bipolar disorder (BD) [85], were checked for overrepresentation in genomic regulatory blocks (Fig. 2). BD-associated loci showed no significant difference from propensity of a random locus to fall in a genomic regulatory block, however, there was a marked increase in ASD-GWAS and SCZ-GWAS detected loci overlapping a genomic regulatory block, indicating a role of genes involved in developmental transcription factors and/or cell adhesion, cell-cell signalling, axon guidance genes (all are enriched among GRB target genes) in molecular mechanisms leading to autism and schizophrenia. It is worth noting here that, since the BD-GWAS study is still in preprint form, no data on the LD blocks were available at the time of writing this analysis and thus the LD block around each SNP was inferred (see Supplementary Methods); updating with actual LD blocks might potentially result in under-/over-representation of BD loci in the GRBs.

## The GRB model provides alternative, biologically plausible long-range targets

A notable example of an SNP involved in long-range regulation of gene expression is a locus obtained by merging two SNPs in LD, with two nearby *VRK2* and *FANCL* genes highlighted as putative targets of these variants. Instead, we propose an alternative target *BCL11A:* according to the GRB model it is a preferred target for long-range regulation by elements from this entire genomic region, including the LD block spanning these two SNPs. *BCL11A* is implicated in the aetiology of the schizophrenia [46], and the phenotypes associated to SNPs in the same LD block fit with the role of the *BCL11A* gene.

*VRK2* encodes a serine/threonine kinase involved in apoptosis and tumour cell growth signalling pathways. A number of SNPs in the region of *VRK2* have previously been associated with schizophrenia, implicating it in the development of the disease [47–49]. There is also evidence that whole blood *VRK2* mRNA levels are lower in schizophrenia patients than healthy controls [48]. *FANCL* is an ubiquitin ligase, involved in DNA repair. *BCL11A* is, however, implicated in brain development, and the haploinsufficient mice display cognition deficits and impaired social behaviour [50].

**Fig. 3:**
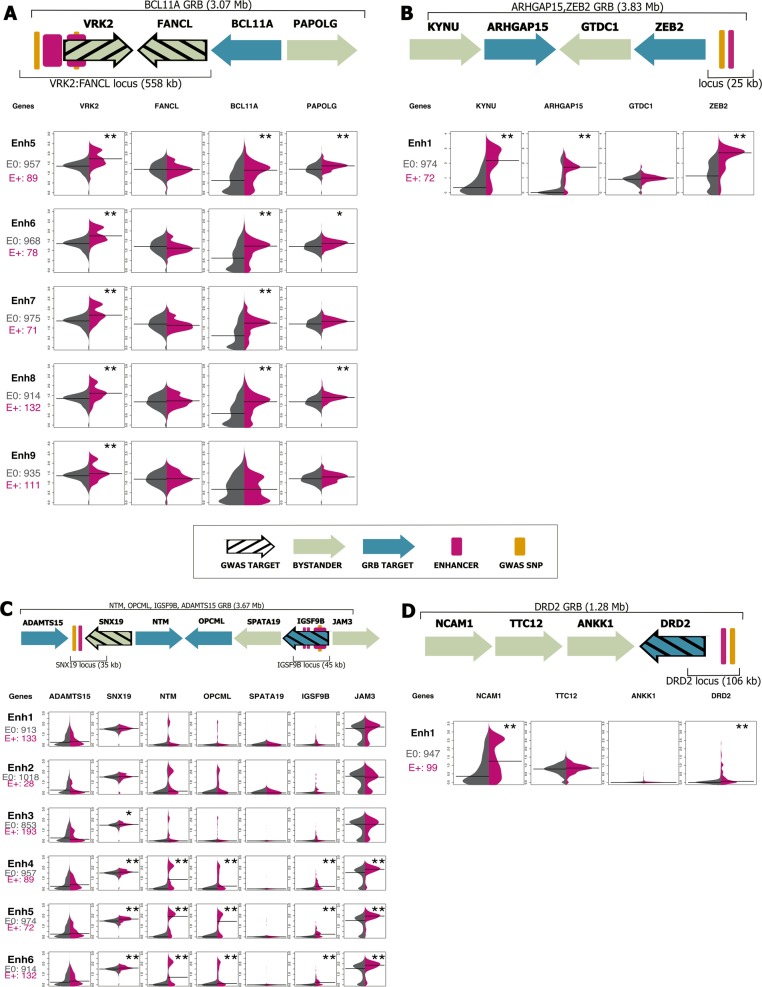
A schematic view of four genomic regulatory blocks. Arrows represent genes, with their orientation indicated by the direction of the arrow. The lower plot in each example is a matrix of log expression values (in TPM) for each enhancer–promoter pair in that GRB. The distribution of expression in tissues where the enhancer is inactive or active is shown in grey and pink, respectively, and the number of samples where each enhancer is (in)active is given under the enhancer label. The black line represents the median expression for each distribution. Significant differences between the medians of the two categories are marked with *p < 0.05, **p < 0.01 and ***p < 0.001 (permutation test, see Supplementary methods). Note, the schematics are not to scale and merely represent gene order (for to-scale plots, visit http://scz.genereg.net/)

This locus is spanned by a GRB whose predicted target is *BCL11A*: a developmental transcription factor essential for cortical development (Fig. 3a). In fact, upon further reanalysis of the schizophrenia-associated variation [42], Basak et al. found SNPs in the second intron of the *BCL11A* gene with significance of association with schizophrenia just missing the genome-wide cut-off at *p* = 1.52e−07 [46]. The activity of enhancer elements Enh5–Enh8 are correlated with the expression of *VRK2*, potentially through short-range regulatory effects, but the dynamic range of the *VRK2*′s response to enhancer activity is small (shown as the change in median expression values between grey and purple distributions in Fig. 3a). However, similar to the example of the obesity linked discussed above [17], there is also a strong positive correlation of elements Enh5–Enh8 with *BCL11A* transcription (despite a 2.5 Mb separation between the gene and the regulatory elements). Due to the extreme distance between this enhancer cluster and the promoter of the *BCL11A* gene, this connection will be missed by any of the methods relying on a fixed genomic cut-off for the enhancer–promoter interactions, even in the most generous case presented in the recent schizophrenia TWAS [23, 24] (max. distance of 500 kb), 2 Mb distance by Huo et al. [26] and in a zebrafish phenotype atlas [25]. In fact, both TWAS analyses reported *FANCL* as a significant hit, but failed to further corroborate the link between this locus and the *FANCL* by the Hi-C data [23], nor the gene list analysis [24].

We propose a scenario in which the whole *VRK/FANCL* region’s chromatin state differs between the two alleles, which can be detectable in TWAS studies and as increased transcriptional activity of the region with two SNPs in the promoter region of the VRK2. However, both of these genes are ubiquitously expressed across tissues, and have a rather large baseline transcriptional activity: these are hallmarks of GRB bystander genes, which are not dependent on activation via long-range enhancers [16]. Moreover, the association of another variant in the VRK2 promoter region, rs2312147, with white matter volume in healthy subjects [45], and white matter connectivity in schizophrenia patients [47] implicates this locus in aberrant brain development. Given *BCL11A*’s role in the regulation of neuronal migration in the developing cortex, and in agreement with the neural network hypothesis of schizophrenia aetiology [38], we conclude that the evidence points to *BCL11A* activation by this enhancer as the most likely biological mechanisms responsible for the GWAS hits at this locus.

In this example, while there is evidence that *VRK2* might play a role in schizophrenia, the variant’s location adjacent to Enh5–Enh8 region suggests that the enhancers in the vicinity of the identified risk variants also regulates *BCL11A*, with larger effect on its expression level than that of *VRK2*. Therefore, the hypothesis-free annotation of GRB targets has provided a more biologically plausible candidate gene at this locus.

## The GRB model predicts the potential targets of unannotated SNPs

An advantage of the GRB model is that it provides an unbiased, systematic list of target genes for all non-coding SNPs within the GRB. This is illustrated by the LD block chr2:146416922–146441832 harbouring a chr2_146436222_I insertion located in a gene desert. This GRB spans the *ZEB2*, *GTDC1*, *ARHGAP15* and *KYNU* genes, with *ARHGAP15* and *ZEB2* being the predicted targets of long-range regulation (Fig. 3b). The expression of both predicted GRB target genes is significantly correlated with the activity of the enhancer overlapping this insertion. *KYNU* expression, which is not found to be responsive to long-range regulation in this locus, is also significantly correlated with the activity of the enhancer. Despite this, the directionality index (which indicates the start/end of a TAD [28]) in this region positions *KYNU* outside of the TAD spanning *ARHGAP15*, *ZEB2* and the LD block with the significantly associated insertion, supporting the GRB model target gene predictions.

*ZEB2* encodes the zinc finger E-box binding protein and is a key regulator of neurogenic and gliogenic processes [51]. Heterozygous *ZEB2* mutations in humans cause Mowat–Wilson syndrome, often associated with structural brain abnormalities and intellectual disability. Statistically significant GWAS signals have been detected in three smaller studies for the *ZEB2* gene: in a 2013 PGC GWAS study predating the dataset analysed here [52], in a Han Chinese population GWAS from 2016 [53] and recently in the GWAS meta-analysis including 40,675 schizophrenia patients that included all the PGC patients, and additional 11,260 cases from the CLOZUK sample [8]. Taken together, the link between ZEB2 and neurological development and phenotypes makes *ZEB2* a plausible candidate gene for schizophrenia. On the other hand, mice mutants for the *ARHGAP15* gene showed cognitive deficits due to impaired neuritogenesis in the hippocampus [54], and a de novo synonymous mutation in this gene was found in a patient with sporadic autism [55], followed by a recent report of a chromatin interaction of a schizophrenia GWAS locus with the promoter of the *ARHGAP15* gene [8].

In this example, the GRB model provides us with two novel, testable potential target genes for a schizophrenia-associated variant, which originally had no associated genes, and were confirmed by other GWAS datasets, and subsequently by chromatin conformation data in post-mortem brains.

## The GRB model identifies mechanistically related SNPs

The next example contains two schizophrenia-associated loci that are both found in the same GRB (chr11:130296827–133970287), and thus most likely affect the same gene. As Fig. 3c shows, this GRB spans six genes, three of which (*NTM*, *OPCML* and *IGSF9B*) are predicted GRB targets. The PGC study proposes *IGSF9B* as the putative target of the rs75059851 SNP identified in the original study due to the location of the SNP in the promoter/first intron of two transcript isoforms of the IGSF9B. The expression of all three of the predicted GRB target genes is significantly greater in tissues in which the enhancers are active, Fig. 2c. In this case all three predicted target genes are neuronal specific cell adhesion molecules involved in neuronal development and thus likely candidate genes. Indeed, OPCML has been previously implicated in the development of schizophrenia by multiple studies in European [56] and Thai [57] populations. In addition, increased levels of an NTM isoform have recently been detected in the dorsolateral prefrontal cortex of schizophrenia patients [58]. More importantly, the concordance of putative enhancers in both schizophrenia-associated loci with genes across the entire GRB (Fig. 3c), including *SNX19*, supports the idea that variants rs10791097 (found just downstream of, and originally thought to be a bystander locus to *SNX19* [42]) and rs75059851 share some mechanistic aspects in the aetiology of the disease, and calls for testing for effects of their interaction despite the large genomic distance between them. This example highlights how the GRBs can serve as functional units in which the effects of multiple SNPs can be considered as potentially interacting.

## The unbiased prediction of GRB target genes identifies potentially overlooked candidate genes at well-studied loci

The final example is a locus that overlaps the gene for the dopamine D2 receptor (*DRD2*)—the target of all licensed antipsychotic therapy drugs [59]. The unpredictability of a patient’s response to antipsychotic therapy, and alternative roles of this locus have been under recent scrutiny [60]. This locus overlaps a GRB containing both *DRD2* and a neural cell adhesion molecule gene, *NCAM1*, shown in Fig. 3d. *NCAM1* has previously been implicated in a number of neuropsychiatric disorders, including schizophrenia [61], and our analysis identifies *NCAM1* as another plausible target, despite not being categorized as a GRB target (it’s predictive value is just below the threshold, see Supplementary information). When active, the predicted enhancer element in the schizophrenia-associated locus affects transcription of both *DRD2* and *NCAM1*, with a more prominent effect on the transcription of the *NCAM1* gene (Supplementary Fig. 1). This locus illustrates the risk of hypothesis-driven target gene search: once a gene, e.g. *DRD2*, expected to play a role in disease aetiology is identified, other putative targets in its vicinity may be overlooked.

## Lessons learned from application of the GRB model to disease-associated genomic loci

Recent approaches to identifying pathways through which non-coding variants lead to neuropsychiatric disorders such as schizophrenia suffer from three major conceptual oversimplifications. First and foremost, despite the wealth of the literature published on complex modes of regulation [17, 62], the practice of assigning non-coding variants to nearby genes is still prevalent. The GRB model allows for the expansion of the search for a target beyond adjacent genes and provides boundaries as to which genes should be included and which should not, but only in the cases where GWAS loci occur in the region of the genome implicated in the long-range regulation. Further, automated GRB target gene prediction provides a shortlist of genes most likely to be under the control of long-range regulation.

Next, epistatic effects between variants have been reported for a range of human complex traits, however systematic approaches to identify pairs of variants displaying epistatic effects suffers from multidimensionality problems and low reproducibility due to high false positive rates (for a review see [63]). GRBs as functional regulatory units may allow us to identify epistatic effects of non-coding variants that fall within the same GRBs (as in the *NTM/OPCML/IGSF9B* example in the Fig. 3c), as this effectively reduces the number of statistical tests required potentially allowing for the detection of modest epistatic effects.

Finally, identification of target genes linked to a given locus is often biased towards genes with functions and pathways previously associated with the trait or disease under investigation, potentially overlooking plausible alternative hypotheses. The evolutionary nature of the GRB model allows for an unbiased approach to identification of potential target genes, potentially identifying novel target genes and new disease mechanisms. Of particular interest is a network of mutually interacting transcription factors involved in neuronal development of cortical layers, predicted as targets in SCZ-GRBs: *BCL11B*, *BCL11A*, *TBR1*, *SATB2* and *FEZF2* (Supplementary Fig. S2). Of these, only *BCL11B* is listed among targets in the PGC study with remainder not detected based on the assignment of non-coding SNPs to the closest gene. GRBs often target genes involved in development, which require complex regulation [16]. Our finding that the non-coding genetic loci associated with schizophrenia and autism, but not bipolar disorder, are significantly more likely to occur in GRBs is thus consistent with other evidence that there is a stronger neurodevelopmental component to these disorders than bipolar [64, 65], and indicates novel potential developmental genes linked to these disorders.

## Other schizophrenia GWAS datasets

Since the conception of this study, several smaller schizophrenia GWAS datasets have emerged [8, 23, 24, 26, 27], with many signals from the 2014 PGC study replicated, and some new loci discovered. The most significant change in these is a notable trend towards functional characterisation of SNPs in view of finding regulatory variants using eQTL information [8, 24, 26, 66], chromatin contacts [8, 27] and transcription factor binding profiles [26]. We have analysed the three largest datasets [8, 26, 67] in the same way as the PGC GWAS represented here (Supplementary Fig. S3), and showed that the GRB target gene prediction still implicates many novel long-range contacts not documented in even the most recent published data (Supplementary information and Supplementary Tables S2 andS3). While a greater coverage of high-resolution tissue-specific chromatin contacts, allele-specific gene expression, transcriptome maps and genome-wide binding profiles for a wider range of transcription factors will partially close this gap in the future, the GRB approach will stand as an elegant method of shortlisting (or adding additional evidence for) genes through which regulatory non-coding variants exert their effects on disease emergence.

## Conclusions

The last few years have seen a step change in the power of GWAS in neuropsychiatric disorders. This has led to large numbers of novel loci being identified, but raises a new challenge: determining the correct gene(s) linked to these loci. The common practice of assigning non-coding loci identified in GWAS to the nearest is likely to be an oversimplification in a substantial proportion of cases. In particular, it neglects the topological organisation of the genome, and the possibility that a locus may be in, or linked to, a non-coding element that regulates a distant gene. New understanding on the characteristics of non-coding elements in highly conserved GRBs, and their link to TADs can be used to identify the potential target genes for loci in GRBs. We applied this knowledge to the loci from recent GWAS in three neuropsychiatric disorders, to show that for two of them, schizophrenia and autism, there was an excess of loci located in GRBs than would be expected by chance. Further analysis showed the potential of this approach to identify novel plausible genes for the schizophrenia, such as *NTM*, *ARHGAP15* and *ZEB2*. This illustrates the potential value of the GRB approach and the need to consider the role of non-coding elements to guide the biological analysis of loci identified by GWAS.

## Supplementary information

Supplementary materials

Supplementary captions

Table S1

Table S2

Table S3

Figure S1

Figure S2

Figure S3
