# Supplementary material for: Understanding the genetics of neuropsychiatric disorders: the potential role of genomic regulatory blocks
Source: Mol Psychiatry. 2019 Oct 15;25(1):6–18. doi: 10.1038/s41380-019-0518-x (PMC6906185; doi:10.1038/s41380-019-0518-x)
Supplement: Supplementary file 1 — Supplementary materials [file 41380_2019_518_MOESM1_ESM.docx]

supplementary materials for the “Understanding the genetics of neuropsychiatric disorders: the potential role of genomic regulatory blocks”

# Supplementary Methods

## Genome assembly.

## All genomic coordinates mentioned in this study refer to *Homo sapiens* genomic assembly hg19.

## Genomic regulatory blocks

## Genomic regulatory blocks (GRBs) between human and mouse were defined as previously described (1). Briefly, highly-conserved non-coding elements are detected satisfying minimum 96% sequence identity over a minimum of 50 consecutive nucleotides between human (hg19) and mouse (mm10) non-coding sequence. The CNE signal was smoothed and regions of high CNE density were detected using a two-state hidden Markov model (HMM) (high vs. low CNE density). Regions of high CNE density were then defined as GRBs, provided they contained at least one protein coding gene and 10 CNEs.

## Target gene prediction

## A random forest classifier was used to detect the genes most likely to be targets of regulation by GRBs (Tan and Lenhard, in preparation). The classifier was trained on a hand-curated set of GRB target and bystander genes (2) and its predictions were based on a set of informative features of GRB target genes. The most important features, as estimated by the model, were the density of non-coding conservation around the gene, the number and total length of CpG islands associated with the gene and the entropy of the gene’s expression. The random forest classifier provides a support value indicating how likely each gene in a GRB is to be under regulation by the GRB (0 being a bystander and 1 being most likely a gene under long range regulation). GRBs tend to regulate a single target gene, however, there are multiple instances in which the classifier predicts more than one target gene. Therefore, we normalized the support values within each GRB to 0-1, and refer to these values as the normalized support values. The normalized support value is defined as

##

$$X_{i\_norm}\text{=}\frac{X_{i} \text{–}X_{min}}{X_{max} \text{– }X_{min}}$$

## where X_i_norm_ is the normalized support value of the gene i, X_i_ is the original support value of the gene i, X_min_ and X_max_ are the minimal and the maximal original support value observed for a gene in this GRB, respectively (Tan and Lenhard, in preparation).

## All genes with a normalized support value greater than 0.6 are defined as putative GRB target genes, yielding a dataset of 1161 potential targets and 3434 bystanders in the human genome.

All GRBs and their predicted targets can be obtained from the “About” section on the http://scz.genereg.net/ page.

## Enhancer definition

## The enhancers considered in each schizophrenia-associated locus are the subset of the permissive enhancers defined in Andersson et al. that overlap the locus boundaries from Ripke et al. i.e. the schizophrenia-associated LD blocks (3). Enhancer’s activity in each tissue is defined based on total CAGE tag count: ‘inactive’ if the normalized tag count is zero, ‘active’ otherwise.

## Gene expression

## CAGE signal was power-law normalized (4) and clustered using distance-based clustering (R package CAGEr, method “distclu”, default parameters). Each protein-coding gene was assigned a transcription start site (TSS) based on pooled CAGE signal from entire FANTOM5 set of tissue/cell type samples in the following way. First, we identify all TSSs within 500nt upstream and 2000nt downstream of the ensemble-annotated gene start. The cluster with the highest pooled expression (in tags per million, TPMs) among all clusters in the promoter is taken as the dominant TSS, and the position with the highest pooled CAGE tag count is taken as a CAGE-based TSS of that gene. Finally each gene gets the normalized tag count (TPMs from http://fantom.gsc.riken.jp/5/datafiles/latest/extra/CAGE_peaks/hg19.cage_peak_phase1and2combined_tpm_ann.osc.txt.gz) assigned as the expression value, for each of the 1046 tissues or cell types.

## Enhancer – promoter activity correlation

## For each GRB, a matrix of correlations between each enhancer in LD with the GWAS SNP, and promoter of each gene in the GRB has been calculated in the following way. For an enhancer, all 1046 samples (tissues or cell types) are classified into enhancer inactive or active categories, based on the zero or more CAGE tags in the enhancer range in the given tissue.

## Then, for each promoter, a (one-tailed) permutation test is performed to test whether the subset of tissues where enhancer is active has a higher median expression than a random subsample of expression values of the same size as this subset, in 10000 iterations, yielding empirical p-value for each promoter-enhancer pair. All p-values are then Bonferonni-corrected for all the tests performed in that GRB, i.e. n(enhancers) X n(promoters).

## This results in a matrix of log-transformed expression distributions (active and inactive, for each enhancer-promoter pair), and an associated adjusted p-value representing how likely it is that the observed correlation between enhancer and promoter activity occurred by chance, as shown in Figure 3.

# inferring ld blocks around bipolar disorder associated gwas snps

At the time of the preparation of this manuscript, the bipolar disorder (BD) study (5) was still under review and therefore available via bioRxiv (https://www.biorxiv.org/content/early/2018/01/24/173062); the available version included only main text and figures, lacking supplementary data. Therefore, in order to overlap an LD block around each BD-associated SNP with the genomic regulatory block dataset, we roughly approximated BD LD blocks, based on the size of LD blocks reported in the autism spectrum disorder GWAS. Each BD LD block is 96,393bp long (i.e. the median size of ASD LD blocks in (6)), and overlaps exactly one of the BD SNPs presented in the table 1 of the Stahl study. Each BD LD block is positioned so that the significantly associated index SNP overlaps a random position within the block.

# ncam1/drd2 locus additional data

As described above, after random forest predictor trained to detect GRB target genes assigns a support value to each gene overlapping a GRB. Normalization is then implemented on each of the GRBs, so that the highest scoring gene in the GRB has a normalized score of 1, and the lowest scoring one has a score of 0. The raw and rescaled support values for all the genes overlapping the four GRBs mentioned in the main text are listed below. The NA values denote genes for which the pipeline does not yield original support value and are hereafter considered to be bystanders.

| Gene name | Original support value | Normalized support value | Classification |
| --- | --- | --- | --- |
| KYNU | NA | NA | NA |
| ARHGAP15 | 0.562 | 1 | Target |
| GTDC1 | 0.052 | 0 | Bystander |
| ZEB2 | 0.440 | 0.761 | Target |
| ADAMTS15 | 0.534 | 0.787 | Target |
| SNX19 | 0.142 | 0 | Bystander |
| NTM | 0.528 | 0.775 | Target |
| OPCML | 0.532 | 0.783 | Target |
| SPATA19 | 0.476 | 0.671 | Target |
| IGSF9B | 0.640 | 1 | Target |
| JAM3 | NA | NA | NA |
| VRK2 | 0.050 | 0 | Bystander |
| FANCL | 0.050 | 0 | Bystander |
| BCL11A | 0.858 | 1 | Target |
| PAPOLG | 0.148 | 0.121 | Bystander |
| NCAM1 | 0.454 | 0.366 | Bystander |
| TTC12 | 0.402 | 0 | Bystander |
| ANKK1 | 0.434 | 0.225 | Bystander |
| DRD2 | 0.544 | 1 | Target |

Of special note is the *NCAM1* gene: after rescaling it misses the target cutoff of 0.6 (chosen for the optimal accuracy of the classifier). However, the raw support value is indicative of this gene fitting the parameters typical of genes under long range regulation. To further corroborate this argument, the gene expression patterns displayed in Supplementary Figure S1 show that *NCAM1*, unlike *DRD2*, is more responsive to enhancer activity that *DRD2*.

# locus-based plots at http://scz.genereg.net/

This resource provides a summary of the GWAS loci discovered by the most recent SCZ GWAS (7) and provides basic information about colocalization with the GRBs on the SNP and locus level (‘All SNPs’ and ‘All loci’ tabs, respectively). Then for each GRB overlapping locus, two summary plots are provided in the ‘Locus summary’ tab: the former is in scale schematic of the GRB spanning the locus and the matrix of enhancer active/inactive promoter transcription levels, for all enhancer-promoter pairs detected for that GRB. For more detailed description of the tracks in the left plot, check the ‘About tab’ and for the examples discussed in this paper search for ’chr2:146416922-146441832’ (*ARHGAP15/ZEB2*), ‘chr11:130714610-130749330’ (*NTM/OPCML/IGSF9B*), ‘chr2:57943593-58502192’ (*BCL11A*) and ‘chr11:113317794-113423994’ (*NCAM1/DRD2*) in the ‘Locus summary’ tab.

# additional Recent schizophrenia GWAS studies

At the time of the preparation of this manuscript, several other smaller schizophrenia GWAS datasets were reported (8–10). These studies show a welcome inclusion of functional datasets during interpretation of loci, and we summarize the implications of the loci associated therein to the discussion of the role of GRBs in GWAS interpretation. Finally, we provide the same summary plots at scz.genereg.net, under the links in the “About” section.

**Pardinas et al.** (8) performed a meta-analysis of the patients in the CLOZUK cohort and the original PGC set of schizophrenia patients, detecting many signals overlapping with the PGC study, and some new ones. They interpreted the 145 loci found significantly associated to regulatory variants not only in the context of the nearest gene, but also including regulatory variants in confirmed long-range chromatin contacts (based on the Hi-C information from corticogenesis stage of brain development (10)) and eQTLs from transcriptomes of post-mortem brain tissues, displaying a similar ratio of loci overlapping GRBs, and completely novel versus overlapping gene target predictions to the original PGC study, shown in Figure S3.

The GRB-based target gene predictions for these loci can be found in Supplementary Table 2 (all loci), and Supplementary Table 3 (just the loci with novel genes predicted by the GRB method). Notably, all four case-study GWAS loci discussed in main text were replicated in this study. First, any link to the *BCL11A* gene was probably missed due to the max. 500Mb distance limit between variant and genes interrogated for the eQTLs. Next, there is an eQTL between the variant the *ZEB2/ARHGAP15* LD block and the nearby ZEB2 gene, and an evidence of long-range physical interaction of this locus with the *ARHGAP1*5 gene, plus one more variant in this GRB for which we also propose these two genes as targets due to significant association of enhancer activity and transcription profiles of these genes. Next, this GWAS detected a third LD block associated to schizophrenia in the *NTM/OPCML/IGSF9B* GRB, and enhancers in it follow a similar behavior of driving expressions of all three predicted target genes in this block. We argue that the concordance of the eQTL and Hi-C contacts will converge more, once the eQTL target genes are considered with a more relaxed distance threshold (e.g. (11)), and a coverage of chromatin contact data is extended to a wider range of relevant tissues.

**Li et al.** (12) presented a dataset of 124 SNPs in 113 schizophrenia GWAS loci (30 not previously observed in the PGC dataset) in Chinese-only and trans-ancestry analysis. The putative target genes were inferred based on a range of criteria: includes nearest gene to the SNP, known disease-associated coding mutation in LD with the index SNP, along with gene prioritization based on DEPICT(13) or SMR(14), and eQTLs. Similar to the Pardinas dataset, all four example schizophrenia loci from this paper were found here: the *BCL11A* locus is prioritized to *VRK2* (the nearest gene); *ZEB2/ARHGAP15* to *TEX41* (the nearest gene); 2 loci for *NTM/IGSF9B/OPCML* locus: first to *IGSF9B* (the nearest gene and DEPICT hit) and second to *SNX19* (the nearest and in eQTL); and finally, *DRD2/NCAM1* locus just to *DRD2* (nearest and DEPICT).

**Huo et al.** (11) focused on transcription-binding disrupting variants using ChIP-seq profiles of 30 transcription factors, and functional annotation, i.e. eQTLs detected in brain-related tissues. We used their dataset of 66 SNPs with eQTLs (Supplemental Data 2), 97 regulatory SNPs with significant eQTLs in CMC, LIBD, or GTEx (Supplemental Data 4) datasets, and 80 regulatory SNPs with significant eQTL genes within broad range of +/-2Mb around the variant in the CMC dataset (Supplemental Data 5). Figure S3 presents how the significant eQTL gene set for all SNPs in a GWAS locus compares to GRB target prediction, where loci overlap a GRB.

Out of our four case-study examples presented in this paper, only the *IGSF9B/NTM/OPCML* locus had been detected to have a significant eQTL in brain tissues, and only for the *IGSF9B* gene. Interestingly, there is a slight increase in number of loci that fall outside of GRBs in Huo subset, compared to each full set of GWAS loci. We speculate this could be a consequence on focusing on a subset of brain-related eQTLs and transcription factor binding in brain tissues, whereas GRB targets tend to be less tissue-specific genes displaying intermediate gene entropy, specific to developmental genes (data not shown). Therefore, it is possible that these two approaches tissue-specific versus non-coding conservation-based one reveal two slightly different modes of regulation leading to schizophrenia.

BIBLIOGRAPHY:

1. Harmston N, Ing-Simmons E, Tan G, Perry M, Merkenschlager M, Lenhard B. Topologically associating domains are ancient features that coincide with Metazoan clusters of extreme noncoding conservation. Nat Commun. 2017;8(1).

2. Akalin A, Fredman D, Arner E, Dong X, Bryne JC, Suzuki H, et al. Transcriptional features of genomic regulatory blocks. Genome Biol. 2009;10(4):1–13.

3. Andersson R, Gebhard C, Miguel-Escalada I, Hoof I, Bornholdt J, Boyd M, et al. An atlas of active enhancers across human cell types and tissues. Nature [Internet]. 2014;507(7493):455–61. Available from: http://www.ncbi.nlm.nih.gov/pubmed/24670763%5Cnhttp://www.nature.com/doifinder/10.1038/nature12787

4. Balwierz PJ, Carninci P, Daub CO, Kawai J, Hayashizaki Y, van Belle W, et al. Methods for analyzing deep sequencing expression data: constructing the human and mouse promoterome with deepCAGE data. Genome Biol [Internet]. 2009;10(7):R79. Available from: http://www.pubmedcentral.nih.gov/articlerender.fcgi?artid=2728533&tool=pmcentrez&rendertype=abstract%5Cnpapers3://publication/doi/10.1186/gb-2009-10-7-r79

5. Stahl E, Forstner A, McQuillin A, Ripke S, PGC BDWG of the, Ophoff R, et al. Genomewide association study identifies 30 loci associated with bipolar disorder. bioRxiv [Internet]. 2017;173062. Available from: https://www.biorxiv.org/content/early/2017/08/08/173062.1

6. Anney RJL, Ripke S, Anttila V, Grove J, Holmans P, Huang H, et al. Meta-analysis of GWAS of over 16,000 individuals with autism spectrum disorder highlights a novel locus at 10q24.32 and a significant overlap with schizophrenia. Mol Autism. 2017;8(1):1–17.

7. Consortium SWG of the PG. Biological insights from 108 schizophrenia-associated genetic loci. Nature [Internet]. 2014;511:421–7. Available from: http://www.nature.com/doifinder/10.1038/nature13595

8. Pardinas AF, Holmans P, Pocklington AJ, Escott-Price V, Carrera N, Legge SE, et al. Common schizophrenia alleles are enriched in mutation- intolerant genes and in regions under strong background selection. Nat Genet. 2018;50(3):381–9.

9. Lam M, Chen C, Li Z, Martin AR, Bryois J. Comparative genetic architectures of schizophrenia in East Asian and European populations. 2018;

10. Won H, de la Torre-Ubieta L, Stein JL, Parikshak NN, Huang J, Opland CK, et al. Chromosome conformation elucidates regulatory relationships in developing human brain. Nature. 2016;538(7626):523–7.

11. Huo Y, Li S, Liu J, Li X, Luo X. Functional genomics reveal gene regulatory mechanisms underlying schizophrenia risk. Nat Commun [Internet]. Springer US; 2019;10. Available from: http://dx.doi.org/10.1038/s41467-019-08666-4

12. Li Z, Chen J, Yu H, He L, Xu Y, Zhang D, et al. Genome-wide association analysis identifies 30 new susceptibility loci for schizophrenia. 2017;49(11).

13. Pers TH, Karjalainen JM, Chan Y, Westra H-J, Wood AR, Yang J, et al. Biological interpretation of genome-wide association studies using predicted gene functions. Nat Commun. 2015;6:5890.

14. Zhu Z, Zhang F, Hu H, Bakshi A, Robinson MR, Powell JE, et al. Integration of summary data from GWAS and eQTL studies predicts complex trait gene targets. Nat Genet. 2016;48(5):481–7.

15. Canovas J, Berndt FA, Sepulveda H, Aguilar R, Veloso FA, Montecino M, et al. The Specification of Cortical Subcerebral Projection Neurons Depends on the Direct Repression of TBR1 by CTIP1/BCL11a. J Neurosci [Internet]. 2015;35(19):7552–64. Available from: http://www.jneurosci.org/cgi/doi/10.1523/JNEUROSCI.0169-15.2015

16. Custo Greig L, Woodworth M, Galazo M, Padmanabhan H, Macklis J. Molecular logic of neocortical projection neuron specification, development and diversity. Nat Rev Neurosci. 2013;14(11).
