# Supplementary material for: Understanding the genetics of neuropsychiatric disorders: the potential role of genomic regulatory blocks
Source: Mol Psychiatry. 2019 Oct 15;25(1):6–18. doi: 10.1038/s41380-019-0518-x (PMC6906185; doi:10.1038/s41380-019-0518-x)
Supplement: Supplementary file 2 — Supplementary captions [file 41380_2019_518_MOESM2_ESM.pdf]

SUPPLEMENTARY FILES

Table S1: A table of all loci from the PGC study (1) specifying the loci intersecting GRBs, and which target genes have been assigned to each locus by the GWAS study and by the GRB method.

Table S2: A table of loci from Pardinas et al. (2) specifying the loci intersecting GRBs, and which target genes have been assigned to each locus by the GWAS study and by the GRB method. Only a subset with completely novel target genes by the GRB method proposed is presented here; for the full set of loci from this dataset, see Supplementary Table S3.

Table S3: A table of all loci from Pardinas et al. (2) specifying the loci intersecting GRBs, and which target genes have been assigned to each locus by the GWAS study and by the GRB method.

Figure S1: Gene expression distributions, in normalized CAGE tags per million, of the genes in the *DRD2/NCAM1* locus in neural and non-neural tissues. Dashed lines show the mean log gene expression for each category. The number of FANTOM5 tissues/cell types in each of the categories is displayed above each distribution.

Figure S2: Regulatory network of transcription factors putatively targeted by the schizophrenia-associated loci. Sources for interactions: 1) (3), 2) (4).

Figure S3: LD blocks from the Pardinas et al. (2), Li et al. (5) and Huo et al. (6) studies overlap with the GRBs. Loci that do not overlap GRBs are shown in grey. Loci in which the predicted GRB target gene was identified as schizophrenia associated in the original GWAS are shown in light red, and the loci in which the GRB model provides novel target gene predictions are shown in dark red. The upper panel presents all loci from the original GWAS study, and the lower panel refers to the subset of loci with functional annotation, used by the Huo et al. (6).

BIBLIOGRAPHY:

1. Consortium SWG of the PG. Biological insights from 108 schizophrenia-associated genetic loci. Nature [Internet]. 2014;511:421–7. Available from: http://www.nature.com/doifinder/10.1038/nature13595
2. Pardinas AF, Holmans P, Pocklington AJ, Escott-Price V, Carrera N, Legge SE, et al. Common schizophrenia alleles are enriched in mutation- intolerant genes and in regions under strong background selection. Nat Genet. 2018;50(3):381–9.
3. Canovas J, Berndt FA, Sepulveda H, Aguilar R, Veloso FA, Montecino M, et al. The Specification of Cortical Subcerebral Projection Neurons Depends on the Direct Repression of TBR1 by CTIP1/BCL11a. J Neurosci [Internet]. 2015;35(19):7552–64. Available from: http://www.jneurosci.org/cgi/doi/10.1523/JNEUROSCI.0169-15.2015
4. Custo Greig L, Woodworth M, Galazo M, Padmanabhan H, Macklis J. Molecular logic of neocortical projection neuron specification, development and diversity. Nat Rev Neurosci. 2013;14(11).
5. Li Z, Chen J, Yu H, He L, Xu Y, Zhang D, et al. Genome-wide association analysis identifies 30 new susceptibility loci for schizophrenia. 2017;49(11).
6. Huo Y, Li S, Liu J, Li X, Luo X. Functional genomics reveal gene regulatory mechanisms underlying schizophrenia risk. Nat Commun [Internet]. Springer US; 2019;10. Available from: http://dx.doi.org/10.1038/s41467-019-08666-4
