# Supplementary material for: Understanding the genetics of neuropsychiatric disorders: the potential role of genomic regulatory blocks
Source: Mol Psychiatry. 2019 Oct 15;25(1):6–18. doi: 10.1038/s41380-019-0518-x (PMC6906185; doi:10.1038/s41380-019-0518-x)
Supplement: Supplementary file 3 — Table S1 [file 41380_2019_518_MOESM3_ESM.docx]

**Supplementary table S1: A table of all loci from the PGC study (2014.) specifying the loci intersecting GRBs, and which target genes have been assigned to each locus by the GWAS study and by the GRB method.**

| **locusCoordinates** | **gwasSNPs** | **gwasGenes** | **inGRBs** | **GRBtargetGenes** | **GRBbystanderGenes** |
| --- | --- | --- | --- | --- | --- |
| chr6:28303247-28712247 | rs115329265 | Locus too broad | FALSE | NA | NA |
| chr1:97792625-98559084 | rs76869799, rs1702294 | DPYD MIR137 (micro-RNA) | TRUE | DPYD | PTBP2,DPYD-AS1,DPYD-IT1,SEC63P1,RPL26P9,DPYD-AS2,MIR137HG,NFU1P2 |
| chr10:104423800-105165583 | rs7907645, rs11191419, rs55833108, chr10_104957618_I | ARL3 AS3MT C10orf32 CNNM2 CYP17A1 INA NT5C2 PCGF6 PDCD11 SFXN2 TAF5 TRIM8 USMG5 WBP1L | TRUE | TRIM8 | SUFU,RN7SL21P,RNU6-43P,ARL3,SFXN2,WBP1L |
| chr12:2321860-2523731 | rs2007044, rs2239063 | CACNA1C | FALSE | NA | NA |
| chr8:143309503-143330533 | rs4129585 | TSNARE1 | FALSE | NA | NA |
| chr4:103146888-103198090 | rs35518360 | SLC39A8 | FALSE | NA | NA |
| chr7:1896096-2190096 | chr7_2025096_I | MAD1L1 | FALSE | NA | NA |
| chr5:60499143-60843543 | rs4391122 | ZSWIM6 | TRUE | SMIM15 | NDUFAF2,ZSWIM6,RPL3P6,C5orf64,RNU6-913P,RN7SKP157 |
| chr12:123448113-123909113 | rs2851447 | ABCB9 ARL6IP4 C12orf65 CDK2AP1 MPHOSPH9 OGFOD2 PITPNM2 RILPL2 SBNO1 SETD8 | FALSE | NA | NA |
| chr2:200715237-200848037 | chr2_200825237_I | AC073043.2 C2orf47 C2orf69 TYW5 | TRUE | SATB2 | PLCL1,RNU7-147P,SATB2-AS1,SEPHS1P6,FTCDNL1 |
| chr15:91416560-91429040 | rs4702 | FES FURIN MAN2A2 | FALSE | NA | NA |
| chr3:36843183-36945783 | rs75968099 | TRANK1 | FALSE | NA | NA |
| chr14:103996234-104184834 | rs12887734 | AL049840.1 APOPT1 BAG5 CKB KLC1 PPP1R13B TRMT61A XRCC3 ZFYVE21 | FALSE | NA | NA |
| chr15:78803032-78926732 | rs8042374, rs190065944 | AC027228.1 AGPHD1 CHRNA3 CHRNA5 CHRNB4 IREB2 PSMA4 | FALSE | NA | NA |
| chr7:110843815-111205915 | rs13240464 | IMMP2L | TRUE | LRRN3 | IMMP2L,DOCK4,DOCK4-AS1 |
| chr11:130714610-130749330 | rs10791097 | SNX19 | TRUE | ADAMTS15,NTM,OPCML,SPATA19,IGSF9B | ADAMTS8,BAK1P2,C11orf44,PPP1R10P1,SNX19,RN7SL167P,RNU6ATAC12P,NTM-IT,RNU6-1182P,OPCML-IT2,OPCML-IT1,MIR4697,JAM3 |
| chr2:185601420-185785420 | rs11693094 | ZNF804A | FALSE | NA | NA |
| chrX:21193266-21570266 | rs1378559 | CNKSR2 | TRUE | KLHL34 | CNKSR2,SMPX |
| chr10:18681005-18770105 | rs7893279 | CACNB2 | FALSE | NA | NA |
| chr12:57428314-57682971 | rs324017, rs12826178 | LRP1 MYO1A NAB2 NDUFA4L2 NXPH4 R3HDM2 SHMT2 STAC3 STAT6 TAC3 TMEM194A | TRUE | MYO1A,NAB2,LRP1,NXPH4,SHMT2,NDUFA4L2,STAC3,INHBC,INHBE,GLI1,ARHGAP9,DDIT3,MBD6,KIF5A,DTX3,ARHGEF25,SLC26A10,B4GALNT1,AGAP2,CDK4,MARCH9,CYP27B1,METTL1 | TAC3,TMEM194A,STAT6,MIR1228,R3HDM2,RNU6-879P,MARS,RNU6-594P,RN7SL312P,MIR616,DCTN2,PIP4K2C,OS9,AGAP2-AS1,TSPAN31,METTL21B,TSFM,AVIL,RNU6-1083P,CTDSP2,MIR26A2,XRCC6BP1,RN7SKP65,RPL21P103,LRIG3,RPS6P22 |
| chr1:73766426-73991366 | rs12129573 | LRRIQ3 * | FALSE | NA | NA |
| chr2:233559301-233753501 | rs6704768 | C2orf82 EFHD1 GIGYF2 KCNJ13 NGEF | FALSE | NA | NA |
| chr11:124610007-124620147 | rs55661361 | ESAM MSANTD2 NRGN VSIG2 | TRUE | SPA17,ESAM,ROBO3 | SIAE,NRGN,VSIG2,MSANTD2,ROBO4,HEPACAM,HEPN1 |
| chr18:52747686-53200117 | chr18_52749216_D, rs78322266, rs9636107 | TCF4 | TRUE | CCDC68 | RAB27B,MAP1LC3P,RNA5SP459,TCF4,MIR4529,RPL21P126 |
| chr11:46342943-46751213 | chr11_46350213_D | AMBRA1 ARHGAP1 ATG13 CHRM4 CKAP5 CREB3L1 DGKZ F2 HARBI1 MDK ZNF408 | TRUE | MDK,CHRM4 | PHF21A,CREB3L1,DGKZ,MIR4688,AMBRA1,MIR3160-1,HARBI1,ATG13 |
| chr3:180588843-181205585 | chr3_180594593_I, rs9841616 | CCDC39 DNAJC19 FXR1 | TRUE | SOX2 | FXR1,DNAJC19,SOX2-OT,RNU6-4P,FAUP2,RPL7AP25,RN7SL703P,RNA5SP150,RN7SKP265,RPL7L1P8 |
| chr20:37361494-37485994 | rs6065094 | ACTR5 PPP1R16B SLC32A1 | FALSE | NA | NA |
| chr2:57943593-58502192 | rs11682175, rs75575209 | FANCL VRK2 | TRUE | BCL11A | VRK2,FANCL,EIF3FP3,LINC01122,RNU6-508P,RNA5SP94,RNU1-32P,MIR4432,RN7SL361P,RNU6-612P,ATP1B3P1,PAPOLG |
| chr15:84661161-85153461 | rs950169 | ADAMTSL3 GOLGA6L4 ZSCAN2 | FALSE | NA | NA |
| chr18:53453389-53804154 | rs72934570, rs715170 | TCF4 * | TRUE | CCDC68 | RAB27B,MAP1LC3P,RNA5SP459,TCF4,MIR4529,RPL21P126 |
| chr2:198148577-198835577 | rs6434928 | ANKRD44 BOLL COQ10B HSPD1 HSPE1 MARS2 PLCL1 RFTN2 SF3B1 | FALSE | NA | NA |
| chr22:41408556-41675156 | rs9607782 | CHADL EP300 L3MBTL2 RANGAP1 | FALSE | NA | NA |
| chr8:111460061-111630761 | rs36068923 | KCNV1 * | FALSE | NA | NA |
| chr3:2532786-2561686 | rs17194490 | CNTN4 | TRUE | CNTN6 | RN7SL120P,RPL23AP39,RPL21P17,RN7SKP144,CNTN4,CNTN4-AS2,DNAJC19P4,CNTN4-AS1,IL5RA |
| chr11:113317794-113423994 | rs2514218 | DRD2 | TRUE | DRD2 | RNU6-44P,RPL23AP62,NCAM1,RNU7-187P,NCAM1-AS1,TTC12,ANKK1,MIR4301 |
| chr11:133808069-133852969 | rs75059851 | IGSF9B | TRUE | ADAMTS15,NTM,OPCML,SPATA19,IGSF9B | ADAMTS8,BAK1P2,C11orf44,PPP1R10P1,SNX19,RN7SL167P,RNU6ATAC12P,NTM-IT,RNU6-1182P,OPCML-IT2,OPCML-IT1,MIR4697,JAM3 |
| chr3:52541105-52903405 | rs2535627 | GLT8D1 GNL3 ITIH1 ITIH3 | TRUE | SEMA3G,TNNC1,NT5DC2 | PHF7,NISCH,STAB1,SMIM4,PBRM1,RNU6-856P,RNU6ATAC16P |
| chr16:29924377-30144877 | rs12691307 | ALDOA ASPHD1 C16orf92 DOC2A FAM57B GDPD3 HIRIP3 INO80E KCTD13 MAPK3 PPP4C SEZ6L2 TAOK2 TBX6 TMEM219 YPEL3 | FALSE | NA | NA |
| chr22:39975317-40016817 | chr22_39987017_D | CACNA1I | FALSE | NA | NA |
| chr3:135807405-136615405 | rs7432375 | MSL2 NCK1 PCCB PPP2R3A SLC35G2 STAG1 | FALSE | NA | NA |
| chr5:151941104-152797656 | rs79212538, rs111294930, rs2973155, rs12522290 | GRIA1 * | FALSE | NA | NA |
| chrX:68377126-68379036 | rs5937157 | PJA1 | TRUE | EFNB1,FAM155B | STARD8,ACTR3P2,SERBP1P1,PJA1,HMGN1P35,LINC00269,CYCSP43,EDA |
| chr17:2095899-2220799 | rs4523957 | SGSM2 SMG6 SRR TSR1 | TRUE | SCARF1,RILP,TLCD2,SERPINF2,RTN4RL1,HIC1,MNT | SLC43A2,RN7SL105P,PRPF8,MIR22HG,WDR81,SERPINF1,SMYD4,RPA1,DPH1,OVCA2,MIR132,MIR212,SMG6,RN7SL624P,SRR,HNRNPA1P16,TSR1,SNORD91B,SNORD91A,SGSM2,METTL16 |
| chr7:86403226-86459326 | rs12704290 | GRM3 | TRUE |  | GRM3 |
| chr15:61831663-61909663 | rs12903146 | VPS14C * | TRUE | RORA | NARG2,CYCSP38,RNA5SP397 |
| chr1:44029384-44128084 | rs11210892 | KDM4A PTPRF | TRUE | PTPRF,ARTN,IPO13,DPH2,ATP6V0B,CCDC24,SLC6A9 | KDM4A,KDM4A-AS1,ST3GAL3,RNU6-1058P,SHMT1P1,B4GALT2 |
| chr19:19374022-19658022 | rs2905426 | CILP2 GATAD2A HAPLN4 MAU2 NCAN NDUFA13 PBX4 SUGP1 TM6SF2 TSSK6 | FALSE | NA | NA |
| chr1:149998890-150242490 | rs140505938 | ANP32E APH1A C1orf51 C1orf54 CA14 OTUD7B PLEKHO1 VPS45 | FALSE | NA | NA |
| chr6:84279922-84407274 | chr6_84280274_D | SNAP91 | FALSE | NA | NA |
| chr1:2372401-2402501 | rs4648845 | PLCH2 | FALSE | NA | NA |
| chr16:13728459-13761359 | rs7405404 | ERCC4 * | FALSE | NA | NA |
| chr7:104598064-105063064 | rs6466055 | MLL5 PUS7 SRPK2 | FALSE | NA | NA |
| chr1:8411184-8638984 | chr1_8424984_D | RERE SLC45A1 | TRUE | ERRFI1,SLC45A1 | DNAJC11,CAMTA1,RNU1-8P,CAMTA1-IT1,VAMP3,PER3,UTS2,TNFRSF9,PARK7,RNU1-7P,RN7SL729P,RNU6-991P,RERE,RPL7P11,RPL7P7 |
| chr12:110723245-110723245 | rs4766428 | ATP2A2 | FALSE | NA | NA |
| chr4:170357552-170646052 | rs10520163 | C4orf27 CLCN3 NEK1 | FALSE | NA | NA |
| chr6:96459651-96459651 | rs117074560 | FUT9 | FALSE | NA | NA |
| chr22:42315744-42689414 | rs1023500, rs6002655 | CENPM CYP2D6 FAM109B NAGA NDUFA6 SEPT3 SHISA8 SMDT1 SREBF2 TCF20 TNFRSF13C WBP2NL | TRUE | NFAM1 | TCF20 |
| chr2:146416922-146441832 | chr2_146436222_I | TRUE | ARHGAP15,ZEB2 | KYNU,MTND6P11,MTND5P24,MTND4P22,MTND3P9,GTDC1,ZEB2-AS1,TEX41,RPL6P5,RNU7-2P,RPL17P12,PABPC1P2 |  |
| chr11:57386294-57682294 | rs9420 | BTBD18 C11orf31 CLP1 CTNND1 MED19 SERPING1 TMX2 YPEL4 ZDHHC5 | TRUE | YPEL4,TMX2-CTNND1,C11orf31,BTBD18 | MIR130A,CLP1,ZDHHC5,MED19,TMX2,CTNND1 |
| chr11:24367320-24412990 | rs11027857 | LUZP2 * | FALSE | NA | NA |
| chr1:30412551-30437271 | rs1498232 |  | FALSE | NA | NA |
| chr7:137039644-137085244 | rs3735025 | DGKI PTN | TRUE | PTN | CHRM2,KRT8P51,DGKI |
| chr9:84630941-84813641 | rs11139497 | TLE1 | TRUE | TLE1 | RPS20P25,RNU6-1035P,RNA5SP287,SPATA31D5P,SPATA31D4,SPATA31D3,SPATA31D2P,SPATA31D1,SPATA31B1,DDX10P2,RPS6P12 |
| chr1:243503719-244002945 | rs10803138, rs77149735, rs14403, chr1_243881945_I | AKT3 SDCCAG8 | TRUE | ZBTB18 | SDCCAG8,MIR4677,AKT3,FABP7P1,AKT3-IT1,RN7SL148P |
| chr15:40566759-40602237 | rs56205728 | ANKRD63 PAK6 PLCB2 | TRUE | PAK6,PLCB2,ANKRD63 | SRP14-AS1,BMF,BUB1B,C15orf56,PLCB2-AS1,LINC00984,C15orf52,RNA5SP392,PHGR1,DISP2,LINC00594,KNSTRN,IVD,BAHD1 |
| chr19:30981643-31039023 | rs2053079 | ZNF536 | TRUE | ZNF536,TSHZ3 | URI1,TAF9P3,RNA5SP471,RNU6-967P,RNA5SP472,ZNF507,DPY19L3 |
| chr5:88581331-88854331 | rs16867576 | MEF2C * | TRUE | MEF2C,CETN3 | TMEM161B,TMEM161B-AS1,RNA5SP187,RPS3AP22,LINC00461,MEF2C-AS1,MIR3660,MBLAC2,POLR3G |
| chr3:17221366-17888266 | rs4330281 | TBC1D5 | TRUE | SATB1 | TBC1D5,PDCL3P3,RAD23BP1,RNU6-138P |
| chr5:137598121-137948092 | rs10043984, rs3849046 | CDC25C CTNNA1 EGR1 ETF1 FAM53C GFRA3 HSPA9 KDM3B REEP2 | TRUE | CDC25C,FAM53C,REEP2,EGR1,ETF1,HSPA9,LRRTM2,SLC23A1,PROB1,SPATA24 | GFRA3,RN7SL682P,KDM3B,RPL7P19,SNORD63,CTNNA1,RN7SL867P,SIL1,RNA5SP194,RNU6-572P,MATR3,SNORA74A,RNA5SP195,RN7SKP64,PAIP2,MZB1,DNAJC18 |
| chr14:99707919-99719219 | rs2693698 | BCL11B | TRUE | BCL11B | RN7SKP108,VRK1,LINC00618,RN7SL710P,C14orf64,RN7SL714P,C14orf177,RPL3P4,SETD3,CCNK,CCDC85C |
| chr14:72417326-72450526 | rs2332700 | AC005477.1 RGS6 | FALSE | NA | NA |
| chr5:45291475-45393775 | rs1501357 | HCN1 | FALSE | NA | NA |
| chr8:60475469-60954469 | rs6984242 | CA8 * | TRUE | TOX | RNU4-50P,RNA5SP267,SLC2A13P1,CA8 |
| chr2:72357335-72368185 | rs3768644 | CYP26B1 | TRUE | CYP26B1 | DYSF,RPS20P10,EXOC6B,RNU2-39P |
| chr11:123394636-123395986 | rs77502336 | GRAMD1B | FALSE | NA | NA |
| chr2:200161422-200309252 | rs6704641 | SATB2 | TRUE | SATB2 | PLCL1,RNU7-147P,SATB2-AS1,SEPHS1P6,FTCDNL1 |
| chr2:193848340-194028340 | rs59979824 | PCGEM1 * | FALSE | NA | NA |
| chr4:176851001-176875801 | rs1106568 | GPM6A | FALSE | NA | NA |
| chr8:4177794-4192544 | rs10503253 | CSMD1 | FALSE | NA | NA |
| chr2:225334096-225467796 | rs11685299 | CUL3 | FALSE | NA | NA |
| chr8:89340626-89753626 | rs7819570 | MMP16 | FALSE | NA | NA |
| chr16:9875519-9970219 | rs9922678 | GRIN2A | TRUE | GRIN2A | IMPDH1P11 |
| chr14:30189985-30190316 | rs2068012 | PRKD1 | TRUE | FOXG1 | C14orf23,RNU6-864P,RNU11-5P,PRKD1,RNU6-1234P |
| chr3:63792650-64004050 | rs832187 | ATXN7 C3orf49 PSMD6 THOC7 | FALSE | NA | NA |
| chr16:67709340-68311340 | rs8044995 | ACD C16orf86 CENPT CTRL DDX28 DPEP2 DPEP3 DUS2L EDC4 ENKD1 ESRP2 GFOD2 LCAT NFATC3 NRN1L NUTF2 PARD6A PLA2G15 PSKH1 PSMB10 RANBP10 SLC12A4 SLC7A6 SLC7A6OS THAP11 TSNAXIP1 | FALSE | NA | NA |
| chr2:149390778-149520178 | chr2_149429178_D | EPC2 | FALSE | NA | NA |
| chr17:17722402-18030202 | rs8082590 | ATPAF2 DRG2 GID4 LRRC48 MYO15A RAI1 SREBF1 TOM1L2 | TRUE | SREBF1 | RAI1,RAI1-AS1,SMCR5,MIR33B,TOM1L2 |
| chr15:70573672-70628872 | rs12148337 | TLE3 * | TRUE | TLE3 | LINC00593,MIR629,RNU6-745P,UACA |
| chr16:58669293-58682833 | rs12325245 | CNOT1 SLC38A7 | FALSE | NA | NA |
| chr8:27412627-27453627 | rs73229090 | CLU EPHX2 | FALSE | NA | NA |
| chrX:5916533-6032733 | rs12845396 | NLGN4X | FALSE | NA | NA |
| chr6:73132701-73171901 | rs1339227 | RIMS1 | FALSE | NA | NA |
| chr7:24619494-24832094 | chr7_24747494_D | DFNA5 MPP6 OSBPL3 | FALSE | NA | NA |
| chr5:109030036-109209066 | rs4388249 | MAN2A1 | TRUE | MAN2A1 | KRT18P42,RN7SKP230,PGAM5P1 |
| chr4:23366403-23443403 | rs215411 | MIR548AJ2 * | TRUE | PPARGC1A,DHX15,SOD3 | GBA3,CDC42P6,RFPL4AP3,MIR573,RN7SL16P,ATP5LP3,HNRNPA1P65,CCDC149,LGI2 |
| chr5:153671057-153688217 | rs11740474 | GALNT10 | FALSE | NA | NA |
| chr11:109285471-109610071 | rs12421382 | C11orf87 | FALSE | NA | NA |
| chr7:110034393-110106693 | rs211829 | IMMP2L * | TRUE | LRRN3 | IMMP2L,DOCK4,DOCK4-AS1 |
| chr12:29905265-29940365 | rs679087 | TMTC1 | FALSE | NA | NA |
| chr7:131539263-131567263 | rs7801375 | PODXL * | TRUE | PLXNA4,LRGUK | CHCHD3,EXOC4,COX5BP3,SLC35B4 |
| chr1:177247821-177300821 | rs6670165 | FAM5B | TRUE | ASTN1,BRINP2,SEC16B | PAPPA2,PTP4A1P7,MIR488,RASAL2-AS1,RASAL2 |
| chr1:207912183-208024083 | rs7523273 | C1orf132 CD46 CR1L | TRUE | PLXNA2 | C1orf132,CD34,RPS26P13,ATP5G2P1,MIR205HG |
| chr20:48114136-48131649 | rs7267348 | KCNB1 PTGIS | FALSE | NA | NA |
| chr12:92243186-92258286 | rs4240748 | C12orf79 * | TRUE | BTG1 | C12orf79,RPL21P106 |
| chr2:162798555-162910255 | rs2909457 | DPP4 SLC4A10 | TRUE | DPP4,GCG,FAP,GCA,KCNH7 | SLC4A10,RPEP5,TIMM8AP1,EIF3EP2,IFIH1,RNA5SP109 |
| chr19:50067499-50135399 | rs56873913 | NOSIP PRR12 PRRG2 RCN3 RRAS SCAF1 | FALSE | NA | NA |
| chr12:103559855-103616655 | rs10860964 | C12orf42 | TRUE | PAH,ASCL1 | IGF1,LINC00485,RNU7-184P,C12orf42 |
| chr5:140023664-140222664 | chr5_140143664_I | AC005609.1 CD14 DND1 HARS HARS2 IK NDUFA2 PCDHA1 PCDHA10 PCDHA2 PCDHA3 PCDHA4 PCDHA5 PCDHA6 PCDHA7 PCDHA8 PCDHA9 TMCO6 WDR55 ZMAT2 | FALSE | NA | NA |
