# Supplementary material for: Understanding the genetics of neuropsychiatric disorders: the potential role of genomic regulatory blocks
Source: Mol Psychiatry. 2019 Oct 15;25(1):6–18. doi: 10.1038/s41380-019-0518-x (PMC6906185; doi:10.1038/s41380-019-0518-x)
Supplement: Supplementary file 4 — Table S2 [file 41380_2019_518_MOESM4_ESM.docx]

**Supplementary table S2: A table of loci from Pardinas et al. (2) specifying the loci intersecting GRBs, and which target genes have been assigned to each locus by the GWAS study and by the GRB method. Only a subset with completely novel target genes by the GRB method proposed is presented here; for the full set of loci from this dataset, see Supplementary Table S3.**

| **locusCoordinates** | **gwasSNPs** | **gwasGenes** | **inGRBs** | **GRBtargetGenes** | **GRBbystanderGenes** | **inPGC** |
| --- | --- | --- | --- | --- | --- | --- |
| chr1:2372397-2402499 | rs4648845 | PLCH2 | FALSE | NA | NA | TRUE |
| chr1:8392592-8701288 | rs34269918 | RERE,SLC45A1 | TRUE | SLC45A1 | DNAJC11,CAMTA1,RNU1-8P,CAMTA1-IT1,VAMP3,PER3,UTS2,TNFRSF9,PARK7,ERRFI1,RNU1-7P,RN7SL729P,RNU6-991P,RERE,RPL7P11,RPL7P7 | TRUE |
| chr1:30428943-30459412 | rs6694545 | LOC101929406 | FALSE | NA | NA | TRUE |
| chr1:44029353-44137257 | rs2970610 | KDM4A,PTPRF | TRUE | PTPRF,ARTN,IPO13,DPH2,ATP6V0B,CCDC24,SLC6A9 | KDM4A,KDM4A-AS1,ST3GAL3,RNU6-1058P,SHMT1P1,B4GALT2 | TRUE |
| chr1:66304167-66333877 | rs12129719 | PDE4B | FALSE | NA | NA | FALSE |
| chr1:73736562-73991651 | rs12129573 | LOC101927295 | FALSE | NA | NA | TRUE |
| chr1:95837686-95889947 | rs6680011 | FLJ31662 | TRUE |  | RNU1-130P,UBE2WP1,EEF1A1P11,RN7SL831P,NDUFS5P2,RPL7P9 | FALSE |
| chr1:97800233-97885249 | rs11165867 | DPYD,DPYD-AS1 | TRUE | DPYD | PTBP2,DPYD-AS1,DPYD-IT1,SEC63P1,RPL26P9,DPYD-AS2,MIR137HG,NFU1P2 | TRUE |
| chr1:98341152-98559093 | rs2660304 | DPYD,MIR137,MIR137HG,MIR2682 | TRUE | DPYD | PTBP2,DPYD-AS1,DPYD-IT1,SEC63P1,RPL26P9,DPYD-AS2,MIR137HG,NFU1P2 | TRUE |
| chr1:149998923-150214166 | rs140505938 | ANP32E,CA14,OTUD7B,PLEKHO1,VPS45 | FALSE | NA | NA | TRUE |
| chr1:173867252-174613056 | rs6701877 | GPR52,RABGAP1L,RC3H1,SERPINC1,ZBTB37 | FALSE | NA | NA | FALSE |
| chr1:177237533-177334782 | rs4650963 | BRINP2 | TRUE | ASTN1,BRINP2 | PAPPA2,PTP4A1P7,MIR488,SEC16B,RASAL2-AS1,RASAL2 | TRUE |
| chr1:190606775-191091298 | rs55770408, rs28374258 | LOC440704 | FALSE | NA | NA | FALSE |
| chr1:200253612-200269903 | rs6678676 | LINC00862 | TRUE | NR5A2 | RNU6-778P,RNU6-716P,RNU6-609P,RNU6-570P,FAM58BP,LINC00862,ZNF281 | FALSE |
| chr1:239198959-239210058 | rs72769124 | CHRM3 | FALSE | NA | NA | FALSE |
| chr1:243503764-243685760 | rs10803138, rs14403 | MIR4677,SDCCAG8,AKT3 | TRUE | ZBTB18 | SDCCAG8,MIR4677,AKT3,FABP7P1,AKT3-IT1,RN7SL148P | TRUE |
| chr2:22621296-22753225 | rs12712510 | LOC102723362 | TRUE | KLHL29 | RN7SL117P,RNA5SP87,RN7SKP27,ATAD2B | FALSE |
| chr2:57950104-58484172 | rs77011057, rs75575209, rs7596038 | FANCL,VRK2 | TRUE | BCL11A | VRK2,FANCL,EIF3FP3,LINC01122,RNU6-508P,RNA5SP94,RNU1-32P,MIR4432,RN7SL361P,RNU6-612P,ATP1B3P1,PAPOLG | TRUE |
| chr2:73161551-73168593 | rs2077586 | EMX1,SFXN5 | TRUE | EMX1,PRADC1,FBXO41,EGR4 | SFXN5,RAB11FIP5,NOTO,SMYD5,CCT7 | FALSE |
| chr2:73552542-73900900 | rs56145559 | ALMS1,ALMS1-IT1,ALMS1P,NAT8 | FALSE | NA | NA | FALSE |
| chr2:145139727-145186749 | rs12991836 | ZEB2 | TRUE | ARHGAP15,ZEB2 | KYNU,MTND6P11,MTND5P24,MTND4P22,MTND3P9,GTDC1,ZEB2-AS1,TEX41,RPL6P5,RNU7-2P,RPL17P12,PABPC1P2 | FALSE |
| chr2:146416874-146441828 | rs56807175 | U80770 | TRUE | ARHGAP15,ZEB2 | KYNU,MTND6P11,MTND5P24,MTND4P22,MTND3P9,GTDC1,ZEB2-AS1,TEX41,RPL6P5,RNU7-2P,RPL17P12,PABPC1P2 | TRUE |
| chr2:185601420-185785791 | rs10196799 | ZNF804A | FALSE | NA | NA | TRUE |
| chr2:198146381-198940251 | rs6434928 | ANKRD44,ANKRD44-IT1,BOLL,COQ10B,HSPD1,HSPE1,HSPE1-MOB4,MARS2,MOB4,PLCL1,RFTN2,SF3B1 | FALSE | NA | NA | TRUE |
| chr2:199908378-200244409 | rs1451488, rs34719143 | SATB2 | TRUE | SATB2 | PLCL1,RNU7-147P,SATB2-AS1,SEPHS1P6,FTCDNL1,RN7SL717P,C2orf69,TYW5,C2orf47,SPATS2L | TRUE |
| chr2:200547937-201309547 | rs76432012, rs2949006, rs200626410, rs1347692 | C2orf47,C2orf69,FTCDNL1,TYW5,SPATS2L | TRUE | SATB2 | PLCL1,RNU7-147P,SATB2-AS1,SEPHS1P6,FTCDNL1,RN7SL717P,C2orf69,TYW5,C2orf47,SPATS2L | TRUE |
| chr2:225334070-225467840 | rs11685299 | CUL3 | FALSE | NA | NA | TRUE |
| chr2:233559312-233790649 | rs4144797 | C2orf82,EFHD1,GIGYF2,KCNJ13,NGEF | FALSE | NA | NA | TRUE |
| chr3:2435850-2540632 | rs35346733 | CNTN4 | TRUE | CNTN6 | RN7SL120P,RPL23AP39,RPL21P17,RN7SKP144,CNTN4,CNTN4-AS2,DNAJC19P4,CNTN4-AS1,IL5RA | TRUE |
| chr3:10800703-10807667 | rs6800435 | LINC00606 | TRUE |  | ATP2B2,ATP2B2-IT1,ATP2B2-IT2,LINC00606,SLC6A11 | FALSE |
| chr3:16845144-16876476 | rs9881798 | PLCL2 | FALSE | NA | NA | FALSE |
| chr3:17221017-17888256 | rs11409090 | TBC1D5 | TRUE | SATB1 | TBC1D5,PDCL3P3,RAD23BP1,RNU6-138P | TRUE |
| chr3:36843149-36945794 | rs75968099 | TRANK1 | FALSE | NA | NA | TRUE |
| chr3:52965713-53175017 | rs1080500 | RFT1,SFMBT1 | FALSE | NA | NA | FALSE |
| chr3:53443354-53538107 | rs312477 | CACNA1D | TRUE |  | CACNA1D | FALSE |
| chr3:60287845-60293004 | rs1353545 | FHIT | TRUE | FEZF2,CADPS | C3orf67,FHIT,NPCDR1,PTPRG,RPL10AP6,RNU2-10P,PTPRG-AS1,C3orf14,RN7SL863P,RNU6-139P | FALSE |
| chr3:63792668-64003983 | rs704373 | ATXN7,C3orf49,PSMD6,PSMD6-AS2,THOC7,THOC7-AS1 | FALSE | NA | NA | TRUE |
| chr3:71481192-71611630 | rs7632921 | FOXP1,MIR1284 | TRUE | GPR27,PROK2 | MITF,RN7SL418P,UQCRHP4,COX6CP6,HMGB1P36,RNU6-281P,FOXP1,FOXP1-AS1,MIR1284,FOXP1-IT1,EIF4E3,RN7SL271P,UBE2Q2P9,LINC00877,LINC00870,RYBP,RNU1-62P | FALSE |
| chr3:135807609-136615268 | rs7432375 | MSL2,NCK1,NCK1-AS1,PCCB,PPP2R3A,SLC35G2,STAG1 | FALSE | NA | NA | TRUE |
| chr3:161394234-161518228 | rs489939 | OTOL1 | TRUE | OTOL1 |  | FALSE |
| chr3:180571624-181207851 | rs34796896, rs55672338 | DNAJC19,FXR1,LOC101928882,SOX2-OT | TRUE | SOX2 | FXR1,DNAJC19,SOX2-OT,RNU6-4P,FAUP2,RPL7AP25,RN7SL703P,RNA5SP150,RN7SKP265,RPL7L1P8 | TRUE |
| chr4:23366446-23443426 | rs215411 | MIR548AJ2 | TRUE | PPARGC1A,DHX15,SOD3 | GBA3,CDC42P6,RFPL4AP3,MIR573,RN7SL16P,ATP5LP3,HNRNPA1P65,CCDC149,LGI2 | TRUE |
| chr4:103001649-103198082 | rs13107325 | BANK1,SLC39A8 | FALSE | NA | NA | TRUE |
| chr4:143742567-143883013 | rs13121251 | INPP4B | FALSE | NA | NA | FALSE |
| chr4:170357792-170646003 | rs10520163 | C4orf27,CLCN3,NEK1 | FALSE | NA | NA | TRUE |
| chr4:176717618-176904037 | rs12498839, rs62334820 | GPM6A | FALSE | NA | NA | TRUE |
| chr5:44960923-45285752 | rs16902086 | HCN1 | FALSE | NA | NA | TRUE |
| chr5:49441779-49884022 | rs77853293 | EMB | FALSE | NA | NA | FALSE |
| chr5:60563907-60843706 | rs7701440 | ZSWIM6 | TRUE | SMIM15 | NDUFAF2,ZSWIM6,RPL3P6,C5orf64,RNU6-913P,RN7SKP157 | TRUE |
| chr5:87676693-88195380 | rs254782 | LINC00461,LOC102546226,MEF2C,MEF2C-AS1,MIR9-2,TMEM161B-AS1 | TRUE | MEF2C,CETN3 | TMEM161B,TMEM161B-AS1,RNA5SP187,RPS3AP22,LINC00461,MEF2C-AS1,MIR3660,MBLAC2,POLR3G | FALSE |
| chr5:88580998-88748452 | rs16867576 | AL050132 | TRUE | MEF2C,CETN3 | TMEM161B,TMEM161B-AS1,RNA5SP187,RPS3AP22,LINC00461,MEF2C-AS1,MIR3660,MBLAC2,POLR3G | TRUE |
| chr5:137838122-137948140 | rs13169274 | ETF1,HSPA9,SNORD63 | TRUE | REEP2,EGR1,LRRTM2,SLC23A1,PROB1,SPATA24 | GFRA3,RN7SL682P,CDC25C,FAM53C,KDM3B,RPL7P19,ETF1,HSPA9,SNORD63,CTNNA1,RN7SL867P,SIL1,RNA5SP194,RNU6-572P,MATR3,SNORA74A,RNA5SP195,RN7SKP64,PAIP2,MZB1,DNAJC18 | TRUE |
| chr5:151924995-152847217 | rs79212538, rs111294930, rs2910032, rs12522290 | LOC101927134 | FALSE | NA | NA | TRUE |
| chr6:73132745-73171881 | rs1339227 | RIMS1 | FALSE | NA | NA | TRUE |
| chr6:83789798-83897565 | rs4470825 | DOPEY1,PGM3,RWDD2A,UBE3D | FALSE | NA | NA | FALSE |
| chr6:84264202-84409255 | rs217287 | SNAP91 | FALSE | NA | NA | TRUE |
| chr6:93063529-93165206 | rs634940 | BC037927 | TRUE | EPHA7 | BACH2,RN7SKP110,MIR4464,MAP3K7,MIR4643,CASC6,RN7SL415P,RPL5P19,ATF1P1,COPS5P1 | FALSE |
| chr6:114679177-114722063 | rs760608 | HS3ST5 | TRUE | HS3ST5 | MARCKS,HDAC2,NUDT19P3,RPSAP43,RNA5SP213,DNAJA1P4 | FALSE |
| chr6:128301981-128333682 | rs35736453 | PTPRK | TRUE | LAMA2 | THEMIS,PTPRK,EEF1DP5,MESTP1,BMPR1APS1,RNU6-861P | FALSE |
| chr6:143645035-143707354 | rs72342102 | AIG1 | TRUE | PEX3 | AIG1,ADAT2,TUBB8P2,RNA5SP221,VDAC1P8,FUCA2 | FALSE |
| chr7:1877502-2190100 | rs10650434 | MAD1L1,MIR4655 | FALSE | NA | NA | TRUE |
| chr7:24719606-24799227 | rs146678232 | DFNA5,MPP6 | FALSE | NA | NA | TRUE |
| chr7:86403263-86948073 | rs12704290, rs147922658 | DMTF1,KIAA1324L,TMEM243,TP53TG1,GRM3 | TRUE |  | GRM3 | TRUE |
| chr7:104597669-105063372 | rs7789569 | KMT2E,KMT2E-AS1,LINC01004,SRPK2 | FALSE | NA | NA | TRUE |
| chr7:110034378-110106697 | rs211829 | IMMP2L | TRUE | LRRN3 | IMMP2L,DOCK4,DOCK4-AS1 | TRUE |
| chr7:110850439-111180544 | rs12705761 | IMMP2L | TRUE | LRRN3 | IMMP2L,DOCK4,DOCK4-AS1 | TRUE |
| chr7:131539274-131627162 | rs7801375, rs4523180 | LOC101928782 | TRUE | PLXNA4,LRGUK | CHCHD3,EXOC4,COX5BP3,SLC35B4 | TRUE |
| chr7:137039670-137085250 | rs3735025 | DGKI,PTN | TRUE | PTN | CHRM2,KRT8P51,DGKI | TRUE |
| chr8:4177791-4208761 | rs139425113 | CSMD1 | FALSE | NA | NA | TRUE |
| chr8:10030698-10057705 | rs11993663 | MSRA | TRUE |  | LINC00599,MSRA | FALSE |
| chr8:18396405-18429406 | rs2410572 | PSD3 | FALSE | NA | NA | FALSE |
| chr8:26190836-26279173 | rs1042992 | BNIP3L,PPP2R2A | FALSE | NA | NA | FALSE |
| chr8:27327841-27453762 | rs2565065, rs11783093 | CHRNA2,PTK2B,CLU,EPHX2,MIR6843 | FALSE | NA | NA | TRUE |
| chr8:34257317-34386259 | rs55669358 | LINC01288 | TRUE | UNC5D | RN7SL457P,VENTXP5,LSM12P1,RN7SKP201,MTND6P19,RNU6-533P,RPL23P10 | FALSE |
| chr8:38014429-38310910 | rs10156310 | ASH2L,BAG4,DDHD2,FGFR1,LETM2,LSM1,PPAPDC1B,STAR,WHSC1L1 | TRUE | LETM2 | WHSC1L1,FGFR1,RPS20P22,C8orf86,RNF5P1 | FALSE |
| chr8:60475926-60954059 | rs1473594 | CA8 | TRUE | TOX | RNU4-50P,RNA5SP267,SLC2A13P1,CA8 | TRUE |
| chr8:89221915-89462854 | rs7010876 | MMP16 | FALSE | NA | NA | TRUE |
| chr8:111460027-111630275 | rs36043959 | KCNV1 | FALSE | NA | NA | TRUE |
| chr8:143276678-143358316 | rs4976967, rs58033671, rs67439964 | LINC00051,MIR4472-1,TSNARE1 | TRUE |  | MIR4472-1,LINC00051,TSNARE1 | TRUE |
| chr9:84607758-84813653 | rs1319017 | SPATA31D1 | TRUE | TLE1 | RPS20P25,RNU6-1035P,RNA5SP287,SPATA31D5P,SPATA31D4,SPATA31D3,SPATA31D2P,SPATA31D1,SPATA31B1,DDX10P2,RPS6P12 | TRUE |
| chr9:101065115-101076627 | rs10985817 | GABBR2 | FALSE | NA | NA | FALSE |
| chr10:18538669-18751891 | rs7099380, rs7893279 | CACNB2 | FALSE | NA | NA | TRUE |
| chr10:104570118-105059896 | rs7476192, rs12416331 | AS3MT,C10orf32,C10orf32-ASMT,CNNM2,CYP17A1,INA,NT5C2,PCGF6,RPEL1,WBP1L | FALSE | NA | NA | TRUE |
| chr11:24367339-24412992 | rs1899543 | LUZP2 | FALSE | NA | NA | TRUE |
| chr11:30240470-30395895 | rs1765142 | ARL14EP,FSHB,MPPED2 | TRUE | MPPED2,PAX6,WT1 | ARL14EP,DCDC1,CYCSP25,DNAJC24,IMMP1L,ELP4,RCN1,EIF4A2P5,WT1-AS,EIF3M,HNRNPA3P9,CCDC73,PRRG4 | FALSE |
| chr11:46342942-46751495 | rs7951870 | AMBRA1,ARHGAP1,ATG13,CHRM4,CKAP5,CREB3L1,DGKZ,F2,HARBI1,MDK,MIR3160-1,MIR3160-2,MIR4688,ZNF408 | TRUE | MDK,CHRM4 | PHF21A,CREB3L1,DGKZ,MIR4688,AMBRA1,MIR3160-1,HARBI1,ATG13 | TRUE |
| chr11:57385856-57681828 | rs7129727 | BTBD18,C11orf31,CLP1,CTNND1,MED19,MIR130A,SERPING1,TMX2,TMX2-CTNND1,YPEL4,ZDHHC5 | TRUE | YPEL4,C11orf31 | MIR130A,CLP1,ZDHHC5,MED19,TMX2,TMX2-CTNND1,BTBD18,CTNND1 | TRUE |
| chr11:65378028-65485218 | rs58950470 | EHBP1L1,KAT5,KCNK7,MAP3K11,MIR4489,MIR4690,PCNXL3,RELA,RNASEH2C,SIPA1 | FALSE | NA | NA | FALSE |
| chr11:113317745-113451229 | rs2514218, rs4936277 | DRD2,MIR4301 | TRUE | DRD2 | RNU6-44P,RPL23AP62,NCAM1,RNU7-187P,NCAM1-AS1,TTC12,ANKK1,MIR4301 | TRUE |
| chr11:124610011-124620147 | rs12293670 | ESAM,MSANTD2,NRGN,VSIG2 | TRUE | ESAM,ROBO3 | SIAE,SPA17,NRGN,VSIG2,MSANTD2,ROBO4,HEPACAM,HEPN1 | TRUE |
| chr11:130794253-130894131 | rs35774874 | SNX19 | TRUE | ADAMTS15,NTM,OPCML,SPATA19,IGSF9B | ADAMTS8,BAK1P2,C11orf44,PPP1R10P1,SNX19,RN7SL167P,RNU6ATAC12P,NTM-IT,RNU6-1182P,OPCML-IT2,OPCML-IT1,MIR4697,JAM3 | TRUE |
| chr11:132387460-132581442 | rs5795787, rs2917569 | OPCML | TRUE | ADAMTS15,NTM,OPCML,SPATA19,IGSF9B | ADAMTS8,BAK1P2,C11orf44,PPP1R10P1,SNX19,RN7SL167P,RNU6ATAC12P,NTM-IT,RNU6-1182P,OPCML-IT2,OPCML-IT1,MIR4697,JAM3 | FALSE |
| chr11:133822133-133853008 | rs4936215 | IGSF9B | TRUE | ADAMTS15,NTM,OPCML,SPATA19,IGSF9B | ADAMTS8,BAK1P2,C11orf44,PPP1R10P1,SNX19,RN7SL167P,RNU6ATAC12P,NTM-IT,RNU6-1182P,OPCML-IT2,OPCML-IT1,MIR4697,JAM3 | TRUE |
| chr11:134290032-134297345 | rs893949 | B3GAT1,LOC283177 | FALSE | NA | NA | FALSE |
| chr12:2321868-2523772 | rs2007044, rs12823424 | CACNA1C,CACNA1C-AS4,CACNA1C-IT3 | FALSE | NA | NA | TRUE |
| chr12:23477601-23637351 | rs1120004 | SOX5 | TRUE | SOX5 | C2CD5,ETNK1,RPS27P22,MIR920,LINC00477,KNOP1P1,RN7SL38P | TRUE |
| chr12:39448519-39533484 | rs10783624 | CPNE8 | FALSE | NA | NA | FALSE |
| chr12:57483524-57682956 | rs324015, rs61937595 | NAB2,STAT6,TMEM194A,LRP1,NDUFA4L2,NXPH4,R3HDM2,SHMT2,STAC3 | TRUE | NAB2,NXPH4,SHMT2,NDUFA4L2,STAC3,ARHGAP9,DDIT3,KIF5A,ARHGEF25,SLC26A10,B4GALNT1,AGAP2,CDK4,MARCH9,CYP27B1 | TAC3,MYO1A,TMEM194A,STAT6,LRP1,MIR1228,R3HDM2,RNU6-879P,INHBC,INHBE,GLI1,MARS,RNU6-594P,RN7SL312P,MIR616,MBD6,DCTN2,PIP4K2C,DTX3,OS9,AGAP2-AS1,TSPAN31,METTL1,METTL21B,TSFM,AVIL,RNU6-1083P,CTDSP2,MIR26A2,XRCC6BP1,RN7SKP65,RPL21P103,LRIG3,RPS6P22 | TRUE |
| chr12:92243186-92258265 | rs4240748 | BTG1 | TRUE | BTG1 | C12orf79,RPL21P106 | TRUE |
| chr12:103361112-103382325 | rs36104021 | ASCL1 | TRUE | ASCL1 | IGF1,LINC00485,PAH,RNU7-184P,C12orf42 | FALSE |
| chr12:110723245-110723245 | rs4766428 | ATP2A2 | FALSE | NA | NA | TRUE |
| chr12:123447928-123902361 | rs2851447 | ABCB9,ARL6IP4,C12orf65,CDK2AP1,LOC100507091,MIR4304,MIR8072,MPHOSPH9,OGFOD2,PITPNM2,RILPL2,SBNO1,SETD8 | FALSE | NA | NA | TRUE |
| chr13:79855297-80162555 | rs9545047 | NDFIP2,NDFIP2-AS1,RBM26,RBM26-AS1 | TRUE | POU4F1 | SLAIN1,MIR3665,EDNRB-AS1,EDNRB,RNF219-AS1,RN7SL810P,LINC01069,LINC00446,SRGNP1,RNY3P3,RPL31P54,TCEB1P23,RNF219,RPL21P111,LINC00331,HSPD1P8,CCT5P2,NIPA2P5,BCAS2P3,RBM26 | FALSE |
| chr14:29469373-29490300 | rs10148671 | FOXG1 | TRUE | FOXG1 | C14orf23,RNU6-864P,RNU11-5P,PRKD1,RNU6-1234P | FALSE |
| chr14:30000405-30190316 | rs1191551, rs199687649 | MIR548AI,PRKD1 | TRUE | FOXG1 | C14orf23,RNU6-864P,RNU11-5P,PRKD1,RNU6-1234P | TRUE |
| chr14:33292743-33309495 | rs34179565 | AKAP6 | TRUE | NPAS3 | AKAP6,EGLN3,EGLN3-AS1 | FALSE |
| chr14:59864362-60033892 | rs150437760 | CCDC175,GPR135,JKAMP,L3HYPDH | FALSE | NA | NA | FALSE |
| chr14:72417326-72450533 | rs2332700 | RGS6 | FALSE | NA | NA | TRUE |
| chr14:99700080-99719219 | rs35604463 | BCL11B | TRUE | BCL11B | RN7SKP108,VRK1,LINC00618,RN7SL710P,C14orf64,RN7SL714P,C14orf177,RPL3P4,SETD3,CCNK,CCDC85C | TRUE |
| chr14:104188920-104537680 | rs10083370, rs80020004 | LINC00637,PPP1R13B,XRCC3,ZFYVE21,ASPG,C14orf2,RD3L,TDRD9 | FALSE | NA | NA | TRUE |
| chr15:40566759-40602256 | rs56282503 | ANKRD63,PAK6,PLCB2 | TRUE | PLCB2,ANKRD63 | SRP14-AS1,BMF,BUB1B,PAK6,C15orf56,PLCB2-AS1,LINC00984,C15orf52,RNA5SP392,PHGR1,DISP2,LINC00594,KNSTRN,IVD,BAHD1 | TRUE |
| chr15:47686081-47686081 | rs281299 | SEMA6D | TRUE | SEMA6D,SLC24A5,CTXN2,SLC12A1 | RN7SKP139,MYEF2,DUT,FBN1 | FALSE |
| chr15:61831680-61909712 | rs12898315 | VPS13C | TRUE | RORA | NARG2,CYCSP38,RNA5SP397 | TRUE |
| chr15:70573650-70618956 | rs12148337 | TLE3 | TRUE | TLE3 | LINC00593,MIR629,RNU6-745P,UACA | TRUE |
| chr15:78802586-78926726 | rs3743078 | CHRNA3,CHRNA5,CHRNB4,HYKK,IREB2,PSMA4 | FALSE | NA | NA | TRUE |
| chr15:82827938-83391537 | rs783540 | ADAMTS7P1,AP3B2,CPEB1,GOLGA6L9,GOLGA6L10,GOLGA6L17P,LOC80154,LOC283692,LOC283693,LOC338963,LOC727751,LOC102724034,RPS17,UBE2Q2P2 | FALSE | NA | NA | FALSE |
| chr15:84703470-85392298 | rs12908161 | ADAMTSL3,ALPK3,DNM1P41,EFTUD1P1,GOLGA2P7,GOLGA6L4,GOLGA6L5P,LINC00933,LOC440300,LOC642423,LOC102724034,LOC103171574,NMB,SCAND2P,SEC11A,UBE2Q2L,UBE2Q2P1,WDR73,ZNF592,ZSCAN2 | FALSE | NA | NA | TRUE |
| chr15:91416550-91429042 | rs17514846 | FES,FURIN,MAN2A2 | FALSE | NA | NA | TRUE |
| chr16:7744180-7744180 | rs12447542 | RBFOX1 | TRUE | RBFOX1 | RNU7-99P,RNU6-457P,RNU6-328P | FALSE |
| chr16:9875513-9960879 | rs7191183 | GRIN2A | TRUE | GRIN2A | IMPDH1P11 | TRUE |
| chr16:13730867-13763942 | rs7499750 | ERCC4 | FALSE | NA | NA | TRUE |
| chr16:24238065-24240725 | rs198160 | PRKCB | TRUE | PRKCB | CACNG3 | FALSE |
| chr16:29924422-30117253 | rs11646127 | ALDOA,ASPHD1,C16orf92,DOC2A,FAM57B,GDPD3,HIRIP3,INO80E,KCTD13,MAPK3,PPP4C,SEZ6L2,TAOK2,TBX6,TMEM219,YPEL3 | FALSE | NA | NA | TRUE |
| chr16:58538662-58684945 | rs42945 | CNOT1,NDRG4,SETD6,SLC38A7,SNORA46,SNORA76A | FALSE | NA | NA | TRUE |
| chr16:63692643-63712719 | rs17465671 |  | TRUE |  | UBE2FP2,RPS15AP34 | FALSE |
| chr16:67708897-68305708 | rs1975802 | ACD,C16orf86,CENPT,CTRL,DDX28,DPEP2,DPEP3,DUS2,EDC4,ENKD1,ESRP2,GFOD2,LCAT,LOC100131303,MIR6773,NFATC3,NRN1L,NUTF2,PARD6A,PLA2G15,PSKH1,PSMB10,RANBP10,RLTPR,SLC7A6,SLC12A4,THAP11,TSNAXIP1 | FALSE | NA | NA | TRUE |
| chr16:71216588-71392062 | rs2161711 | CALB2,CMTR2,HYDIN | FALSE | NA | NA | FALSE |
| chr17:2059961-2216258 | rs7216638 | LOC101927839,SMG6,SNORD91A,SNORD91B,SRR,TSR1 | TRUE | SCARF1,RILP,TLCD2,RTN4RL1,HIC1 | SLC43A2,RN7SL105P,PRPF8,MIR22HG,WDR81,SERPINF2,SERPINF1,SMYD4,RPA1,DPH1,OVCA2,MIR132,MIR212,SMG6,RN7SL624P,SRR,HNRNPA1P16,TSR1,SNORD91B,SNORD91A,SGSM2,MNT,METTL16 | TRUE |
| chr17:17649172-17967397 | rs4925114 | ATPAF2,GID4,LRRC48,MIR33B,MIR6777,RAI1,SMCR5,SREBF1,TOM1L2 | TRUE | SREBF1 | RAI1,RAI1-AS1,SMCR5,MIR33B,TOM1L2 | TRUE |
| chr17:19014831-19183427 | rs66885728 | EPN2,EPN2-AS1,EPN2-IT1,GRAPL,LOC79999,LOC388436 | FALSE | NA | NA | FALSE |
| chr17:78557051-78685626 | rs7225476 | RPTOR | FALSE | NA | NA | FALSE |
| chr18:52747689-53804156 | rs5825114, rs79926379, rs66791238, rs28758902, rs144158419, rs1789595 | TCF4,LOC101927273,LOC100505474 | TRUE | CCDC68 | RAB27B,MAP1LC3P,RNA5SP459,TCF4,MIR4529,RPL21P126 | TRUE |
| chr18:77566535-77579812 | rs56775891 | KCNG2 | FALSE | NA | NA | FALSE |
| chr19:11849736-11849736 | rs72986630 | ZNF823 | FALSE | NA | NA | FALSE |
| chr19:19358332-19657632 | rs2905432 | CILP2,GATAD2A,HAPLN4,MAU2,NCAN,NDUFA13,PBX4,SUGP1,TM6SF2,TSSK6,YJEFN3 | FALSE | NA | NA | TRUE |
| chr19:30981639-31038995 | rs2053079 | ZNF536 | TRUE | ZNF536,TSHZ3 | URI1,TAF9P3,RNA5SP471,RNU6-967P,RNA5SP472,ZNF507,DPY19L3 | TRUE |
| chr19:50106208-50182697 | rs7508148 | ADM5,BCL2L12,CPT1C,IRF3,MIR5088,PRMT1,PRR12,PRRG2,RRAS,SCAF1 | FALSE | NA | NA | TRUE |
| chr20:20817745-20830612 | rs6035706 | RALGAPA2 | FALSE | NA | NA | FALSE |
| chr20:37361504-37485986 | rs6065094 | ACTR5,PPP1R16B,SLC32A1 | FALSE | NA | NA | TRUE |
| chr22:39840130-40091818 | rs9611177, rs5757730, rs4820386 | LOC100506472,MGAT3,TAB1,CACNA1I,RPS19BP1 | FALSE | NA | NA | TRUE |
| chr22:41408754-41684093 | rs9607782 | CHADL,EP300,EP300-AS1,L3MBTL2,MIR1281,MIR6889,RANGAP1,ZC3H7B | FALSE | NA | NA | TRUE |
| chr22:42315790-42689370 | rs1023497, rs6002655 | CENPM,LINC00634,MIR33A,SEPT3,SHISA8,SREBF2,TNFRSF13C,CYP2D6,CYP2D7P,FAM109B,LOC388906,LOC101929829,NAGA,NDUFA6,NDUFA6-AS1,SEPT3,SMDT1,TCF20,WBP2NL | TRUE | NFAM1 | TCF20 | TRUE |
| chrX:5859733-6029533 | rs12009217 | NLGN4X | FALSE | NA | NA | TRUE |
| chrX:68377126-68377205 | rs62606711 | PJA1 | TRUE | EFNB1,FAM155B | STARD8,ACTR3P2,SERBP1P1,PJA1,HMGN1P35,LINC00269,CYCSP43,EDA | TRUE |
