# Supplementary material for: Understanding the genetics of neuropsychiatric disorders: the potential role of genomic regulatory blocks
Source: Mol Psychiatry. 2019 Oct 15;25(1):6–18. doi: 10.1038/s41380-019-0518-x (PMC6906185; doi:10.1038/s41380-019-0518-x)
Supplement: Supplementary file 5 — Table S3 [file 41380_2019_518_MOESM5_ESM.docx]

**Supplementary table S3: A table of all loci from Pardinas et al. (2) specifying the loci intersecting GRBs, and which target genes have been assigned to each locus by the GWAS study and by the GRB method.**

| **locusCoordinates** | **gwasSNPs** | **gwasGenes** | **GRBtargetGenes** | **GRBbystanderGenes** | **Study** |
| --- | --- | --- | --- | --- | --- |
| chr1:200253612-200269903 | rs6678676 | LINC00862 | NR5A2 | RNU6-778P,RNU6-716P,RNU6-609P,RNU6-570P,FAM58BP,LINC00862,ZNF281 | Pardinas |
| chr1:243503764-243685760 | rs10803138, rs14403 | MIR4677,SDCCAG8,AKT3 | ZBTB18 | SDCCAG8,MIR4677,AKT3,FABP7P1,AKT3-IT1,RN7SL148P | PGC, Pardinas |
| chr2:22621296-22753225 | rs12712510 | LOC102723362 | KLHL29 | RN7SL117P,RNA5SP87,RN7SKP27,ATAD2B | Pardinas |
| chr2:57950104-58484172 | rs77011057, rs75575209, rs7596038 | FANCL,VRK2 | BCL11A | VRK2,FANCL,EIF3FP3,LINC01122,RNU6-508P,RNA5SP94,RNU1-32P,MIR4432,RN7SL361P,RNU6-612P,ATP1B3P1,PAPOLG | PGC, Pardinas |
| chr2:146416874-146441828 | rs56807175 | U80770 | ARHGAP15,ZEB2 | KYNU,MTND6P11,MTND5P24,MTND4P22,MTND3P9,GTDC1,ZEB2-AS1,TEX41,RPL6P5,RNU7-2P,RPL17P12,PABPC1P2 | PGC, Pardinas |
| chr2:200547937-201309547 | rs76432012, rs2949006, rs200626410, rs1347692 | C2orf47,C2orf69,FTCDNL1,TYW5,SPATS2L | SATB2 | PLCL1,RNU7-147P,SATB2-AS1,SEPHS1P6,FTCDNL1,RN7SL717P,C2orf69,TYW5,C2orf47,SPATS2L | PGC, Pardinas |
| chr3:2435850-2540632 | rs35346733 | CNTN4 | CNTN6 | RN7SL120P,RPL23AP39,RPL21P17,RN7SKP144,CNTN4,CNTN4-AS2,DNAJC19P4,CNTN4-AS1,IL5RA | PGC, Pardinas |
| chr3:17221017-17888256 | rs11409090 | TBC1D5 | SATB1 | TBC1D5,PDCL3P3,RAD23BP1,RNU6-138P | PGC, Pardinas |
| chr3:60287845-60293004 | rs1353545 | FHIT | FEZF2,CADPS | C3orf67,FHIT,NPCDR1,PTPRG,RPL10AP6,RNU2-10P,PTPRG-AS1,C3orf14,RN7SL863P,RNU6-139P | Pardinas |
| chr3:71481192-71611630 | rs7632921 | FOXP1,MIR1284 | GPR27,PROK2 | MITF,RN7SL418P,UQCRHP4,COX6CP6,HMGB1P36,RNU6-281P,FOXP1,FOXP1-AS1,MIR1284,FOXP1-IT1,EIF4E3,RN7SL271P,UBE2Q2P9,LINC00877,LINC00870,RYBP,RNU1-62P | Pardinas |
| chr3:180571624-181207851 | rs34796896, rs55672338 | DNAJC19,FXR1,LOC101928882,SOX2-OT | SOX2 | FXR1,DNAJC19,SOX2-OT,RNU6-4P,FAUP2,RPL7AP25,RN7SL703P,RNA5SP150,RN7SKP265,RPL7L1P8 | PGC, Pardinas |
| chr4:23366446-23443426 | rs215411 | MIR548AJ2 | PPARGC1A,DHX15,SOD3 | GBA3,CDC42P6,RFPL4AP3,MIR573,RN7SL16P,ATP5LP3,HNRNPA1P65,CCDC149,LGI2 | PGC, Pardinas |
| chr5:60563907-60843706 | rs7701440 | ZSWIM6 | SMIM15 | NDUFAF2,ZSWIM6,RPL3P6,C5orf64,RNU6-913P,RN7SKP157 | PGC, Pardinas |
| chr5:88580998-88748452 | rs16867576 | AL050132 | MEF2C,CETN3 | TMEM161B,TMEM161B-AS1,RNA5SP187,RPS3AP22,LINC00461,MEF2C-AS1,MIR3660,MBLAC2,POLR3G | PGC, Pardinas |
| chr5:137838122-137948140 | rs13169274 | ETF1,HSPA9,SNORD63 | REEP2,EGR1,LRRTM2,SLC23A1,PROB1,SPATA24 | GFRA3,RN7SL682P,CDC25C,FAM53C,KDM3B,RPL7P19,ETF1,HSPA9,SNORD63,CTNNA1,RN7SL867P,SIL1,RNA5SP194,RNU6-572P,MATR3,SNORA74A,RNA5SP195,RN7SKP64,PAIP2,MZB1,DNAJC18 | PGC, Pardinas |
| chr6:93063529-93165206 | rs634940 | BC037927 | EPHA7 | BACH2,RN7SKP110,MIR4464,MAP3K7,MIR4643,CASC6,RN7SL415P,RPL5P19,ATF1P1,COPS5P1 | Pardinas |
| chr6:128301981-128333682 | rs35736453 | PTPRK | LAMA2 | THEMIS,PTPRK,EEF1DP5,MESTP1,BMPR1APS1,RNU6-861P | Pardinas |
| chr6:143645035-143707354 | rs72342102 | AIG1 | PEX3 | AIG1,ADAT2,TUBB8P2,RNA5SP221,VDAC1P8,FUCA2 | Pardinas |
| chr7:110034378-110106697 | rs211829 | IMMP2L | LRRN3 | IMMP2L,DOCK4,DOCK4-AS1 | PGC, Pardinas |
| chr7:110850439-111180544 | rs12705761 | IMMP2L | LRRN3 | IMMP2L,DOCK4,DOCK4-AS1 | PGC, Pardinas |
| chr7:131539274-131627162 | rs7801375, rs4523180 | LOC101928782 | PLXNA4,LRGUK | CHCHD3,EXOC4,COX5BP3,SLC35B4 | PGC, Pardinas |
| chr8:34257317-34386259 | rs55669358 | LINC01288 | UNC5D | RN7SL457P,VENTXP5,LSM12P1,RN7SKP201,MTND6P19,RNU6-533P,RPL23P10 | Pardinas |
| chr8:60475926-60954059 | rs1473594 | CA8 | TOX | RNU4-50P,RNA5SP267,SLC2A13P1,CA8 | PGC, Pardinas |
| chr9:84607758-84813653 | rs1319017 | SPATA31D1 | TLE1 | RPS20P25,RNU6-1035P,RNA5SP287,SPATA31D5P,SPATA31D4,SPATA31D3,SPATA31D2P,SPATA31D1,SPATA31B1,DDX10P2,RPS6P12 | PGC, Pardinas |
| chr11:130794253-130894131 | rs35774874 | SNX19 | ADAMTS15,NTM,OPCML,SPATA19,IGSF9B | ADAMTS8,BAK1P2,C11orf44,PPP1R10P1,SNX19,RN7SL167P,RNU6ATAC12P,NTM-IT,RNU6-1182P,OPCML-IT2,OPCML-IT1,MIR4697,JAM3 | PGC, Pardinas |
| chr13:79855297-80162555 | rs9545047 | NDFIP2,NDFIP2-AS1,RBM26,RBM26-AS1 | POU4F1 | SLAIN1,MIR3665,EDNRB-AS1,EDNRB,RNF219-AS1,RN7SL810P,LINC01069,LINC00446,SRGNP1,RNY3P3,RPL31P54,TCEB1P23,RNF219,RPL21P111,LINC00331,HSPD1P8,CCT5P2,NIPA2P5,BCAS2P3,RBM26 | Pardinas |
| chr14:30000405-30190316 | rs1191551, rs199687649 | MIR548AI,PRKD1 | FOXG1 | C14orf23,RNU6-864P,RNU11-5P,PRKD1,RNU6-1234P | PGC, Pardinas |
| chr14:33292743-33309495 | rs34179565 | AKAP6 | NPAS3 | AKAP6,EGLN3,EGLN3-AS1 | Pardinas |
| chr15:61831680-61909712 | rs12898315 | VPS13C | RORA | NARG2,CYCSP38,RNA5SP397 | PGC, Pardinas |
| chr17:2059961-2216258 | rs7216638 | LOC101927839,SMG6,SNORD91A,SNORD91B,SRR,TSR1 | SCARF1,RILP,TLCD2,RTN4RL1,HIC1 | SLC43A2,RN7SL105P,PRPF8,MIR22HG,WDR81,SERPINF2,SERPINF1,SMYD4,RPA1,DPH1,OVCA2,MIR132,MIR212,SMG6,RN7SL624P,SRR,HNRNPA1P16,TSR1,SNORD91B,SNORD91A,SGSM2,MNT,METTL16 | PGC, Pardinas |
| chr18:52747689-53804156 | rs5825114, rs79926379, rs66791238, rs28758902, rs144158419, rs1789595 | TCF4,LOC101927273,LOC100505474 | CCDC68 | RAB27B,MAP1LC3P,RNA5SP459,TCF4,MIR4529,RPL21P126 | PGC, Pardinas |
| chr22:42315790-42689370 | rs1023497, rs6002655 | CENPM,LINC00634,MIR33A,SEPT3,SHISA8,SREBF2,TNFRSF13C,CYP2D6,CYP2D7P,FAM109B,LOC388906,LOC101929829,NAGA,NDUFA6,NDUFA6-AS1,SEPT3,SMDT1,TCF20,WBP2NL | NFAM1 | TCF20 | PGC, Pardinas |
| chrX:68377126-68377205 | rs62606711 | PJA1 | EFNB1,FAM155B | STARD8,ACTR3P2,SERBP1P1,PJA1,HMGN1P35,LINC00269,CYCSP43,EDA | PGC, Pardinas |
