# Supplementary figures and images for: Understanding the genetics of neuropsychiatric disorders: the potential role of genomic regulatory blocks
Source: Mol Psychiatry. 2019 Oct 15;25(1):6–18. doi: 10.1038/s41380-019-0518-x (PMC6906185; doi:10.1038/s41380-019-0518-x)

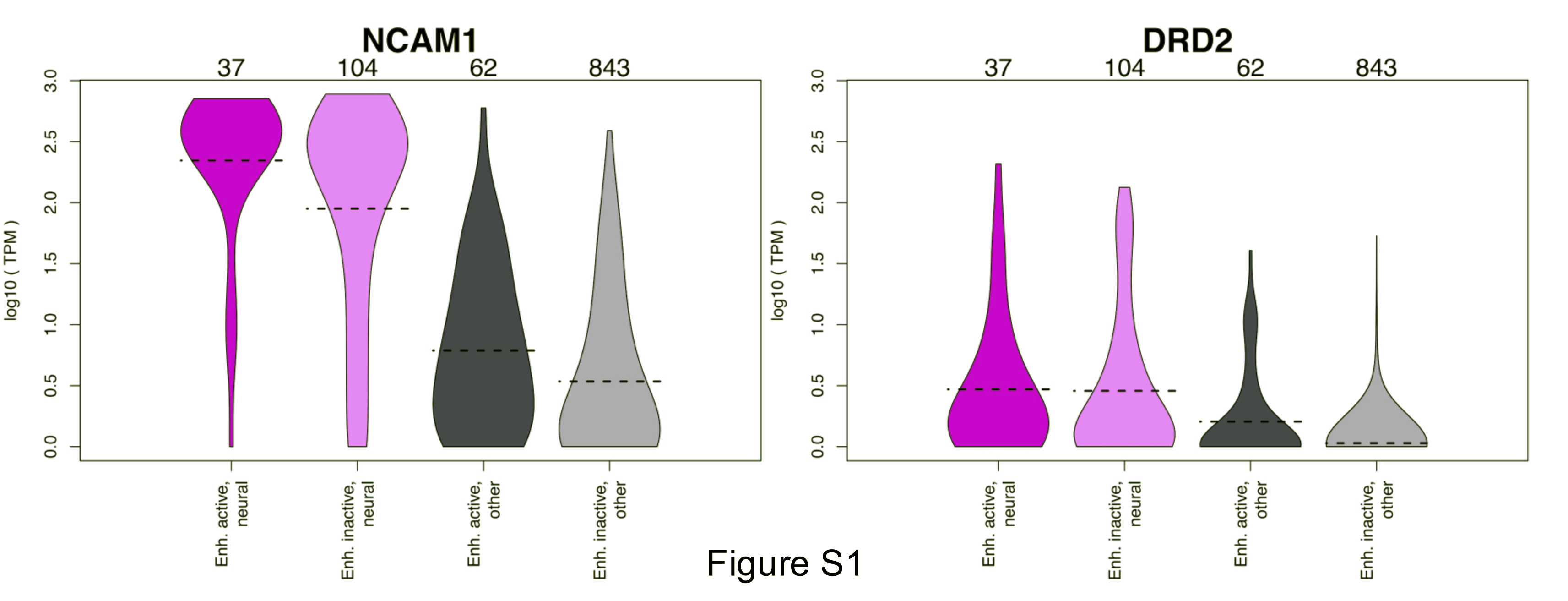

Supplement: Supplementary file 6 — Figure S1 [file 41380_2019_518_MOESM6_ESM.jpg]

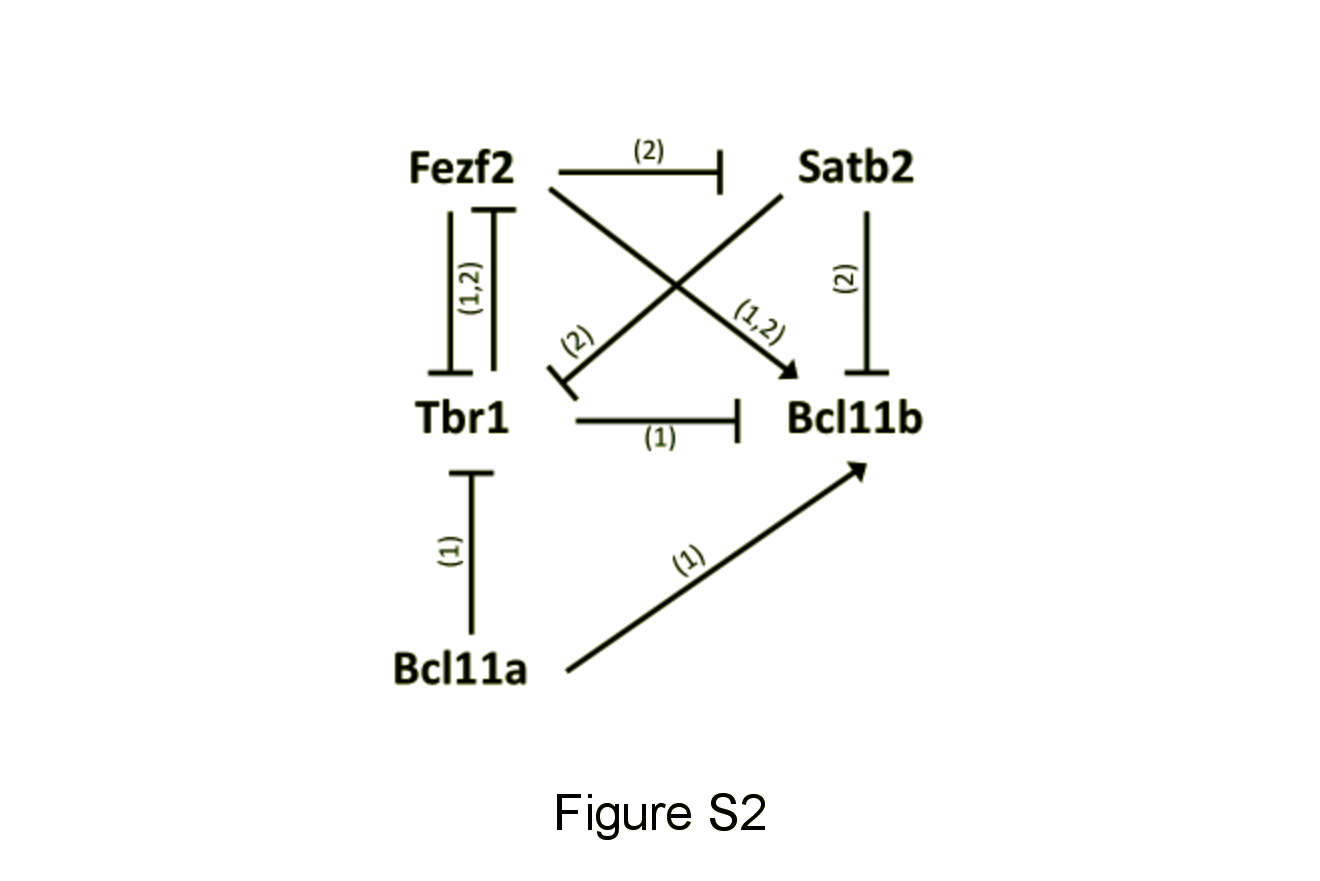

Supplement: Supplementary file 7 — Figure S2 [file 41380_2019_518_MOESM7_ESM.jpg]

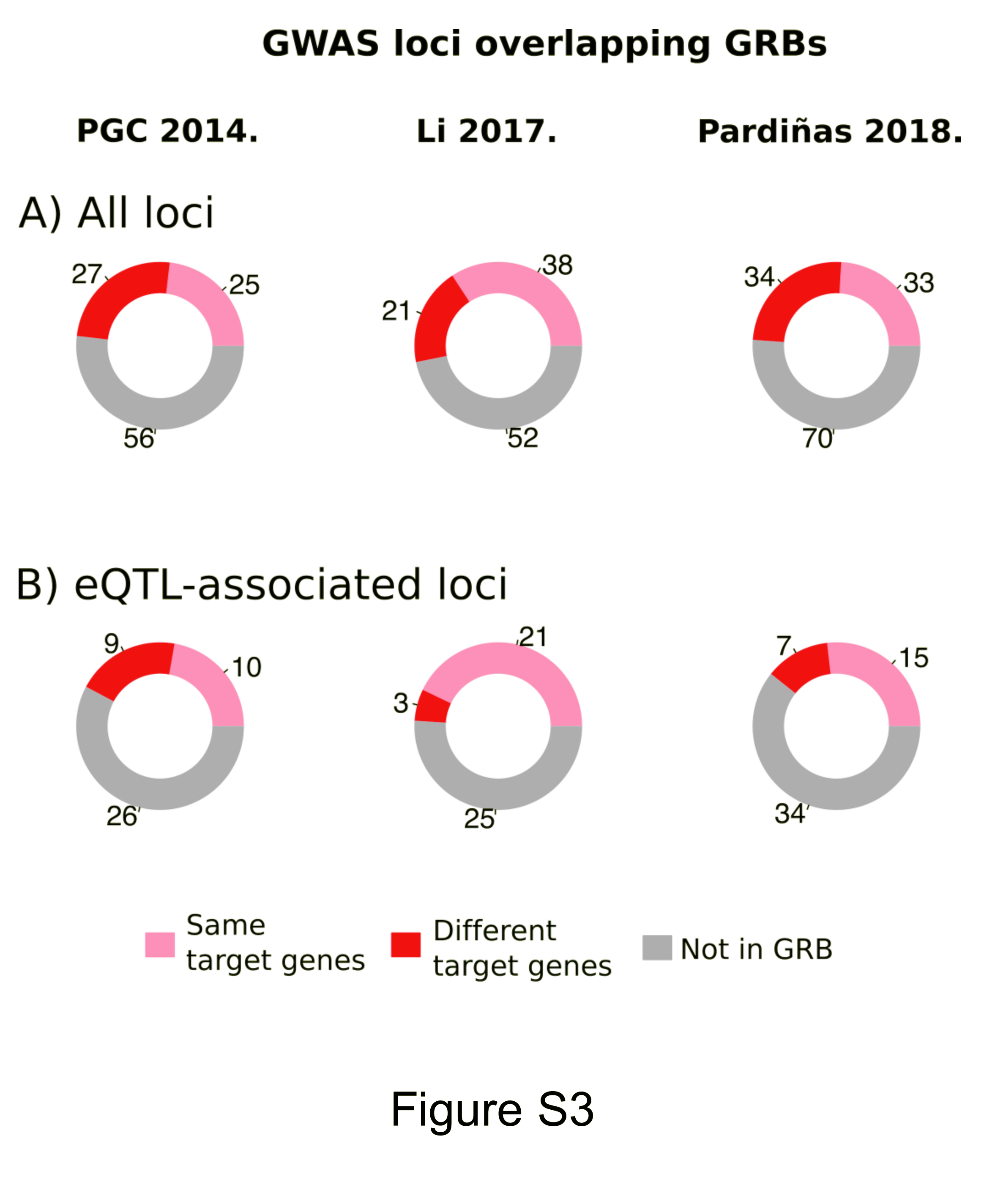

Supplement: Supplementary file 8 — Figure S3 [file 41380_2019_518_MOESM8_ESM.jpg]
